# Predicting Survival in Mucinous Adenocarcinoma of the Appendix: Demographics, Disease Presentation, and Treatment Methodology

**DOI:** 10.1245/s10434-024-15526-z

**Published:** 2024-06-14

**Authors:** Paul H. McClelland, Stephanie N. Gregory, Shirley K. Nah, Jonathan M. Hernandez, Jeremy L. Davis, Andrew M. Blakely

**Affiliations:** Center for Cancer Research, National Cancer Institute, National Institutes of Health, Bethesda, MD USA

## Abstract

**Background:**

Mucinous adenocarcinoma of the appendix (MACA) follows a complex disease course with variable survival. Large-scale predictive modeling may determine subtle yet important prognostic factors otherwise unseen in smaller cohort analyses.

**Methods:**

Patients with MACA were identified from the Surveillance, Epidemiology, and End Results (SEER) Research Plus database (2005–2019). Primary, secondary, and tertiary outcomes were disease-specific survival (DSS), overall survival (OS), and average annual percent change (AAPC) in incidence.

**Results:**

Among 4,258 included patients, MACA was most frequently diagnosed at 50 to 69 years (52.0%), with female preponderance (55.9%). MACA incidence AAPC was 3.8 (95% confidence interval [CI] 1.9–5.9). For patients with exclusive, first-diagnosis MACA included in survival analysis (3,222 patients), median DSS and OS were 118 and 88 months, respectively. In DSS-based multivariable analysis, worse prognosis was associated with non-Hispanic Black background (HR 1.36, 95% CI 1.02–1.82; *p* = 0.036), high grade (grade 3 HR 3.10, 95% CI 2.44–3.92; *p* < 0.001), lymphatic spread (HR 2.73, 95% CI 2.26–3.30; *p* < 0.001), and distant metastasis (HR 5.84, 95% CI 3.86–8.83; *p* < 0.001). In subcohort analysis of patients with rationale for cytoreductive surgery and hyperthermic intraperitoneal chemotherapy (CRS-HIPEC, 2,387 patients), CRS-HIPEC was associated with survival benefit compared with surgery alone but only for moderate-grade tumors (median DSS/OS 138/138 vs. 116/87 months; *p* < 0.001).

**Conclusions:**

Mucinous adenocarcinoma of the appendix incidence is increasing in the United States. Survival rates are affected by both demographics and classical risk factors, and CRS-HIPEC-associated survival benefit predominantly occurs in moderate-grade tumors. Further exploration of biologic and clinicopathologic features may enhance risk stratification for this disease.

**Supplementary Information:**

The online version contains supplementary material available at 10.1245/s10434-024-15526-z.

Mucinous adenocarcinoma of the appendix (MACA), which comprises approximately 40% of appendiceal tumors, represents a complex and challenging clinical problem with a highly variable disease course.^1–3^ Five-year overall survival of MACA is estimated at 54%,^4^ but individual survival depends on multiple clinical predictors, including tumor biology, degree of invasion, presence of *pseudomyxoma peritonei *(PMP), and end-organ involvement.^3,5–10^ Accordingly, management of MACA often is individualized and relies on a combination of surgical and systemic interventions, which can vary depending on the clinical indication.^9,10^ Moreover, as novel life-prolonging therapies such as cytoreductive surgery and hyperthermic intraperitoneal chemotherapy (CRS-HIPEC) have emerged over the past two decades, survival rates for MACA have further shifted, and clinical decision-making at the time of diagnosis has become increasingly complicated.^6,11,12^

Because of the rarity of MACA and the logistical complexity of CRS-HIPEC, advanced surgical management of MACA has mostly been performed at specialized centers for select patient populations.^3,9,10^ As such, data demonstrating the efficacy of CRS-HIPEC have largely been derived from single-center cohort analyses or, less commonly, from multicenter collaborations.^6,8,13–20^ Large-scale data may provide additional insight into the efficacy of interventions such as CRS-HIPEC, although such analyses are uncommon.^3,5,21,22^ Moreover, while classical indicators of aggressive or advanced disease such as tumor grade, lymphatic spread, and distant metastasis have been broadly associated with worse outcomes in MACA, other potentially influential factors such as patient demographics, clinical presentation, and overall treatment strategy have not yet been thoroughly explored.^1,2,4^

To this end, this study uses population-level data to analyze the incidence and long-term survival of patients with MACA, using disease-specific survival (DSS) and overall survival (OS) as primary and secondary endpoints. By contextualizing the effects of modern treatment strategies among broader trends in demographics and disease presentation, this analysis seeks to provide a data-driven framework to enhance management strategies in this unique patient population.

## Methods

### Database Selection and Inclusion Criteria

National data were analyzed from the Surveillance, Epidemiology, and End Results (SEER) Research Plus Database to determine trends in patient demographics, disease characteristics, treatment variables, and survival (17 registries, November 2022 submission). A date range of 2005–2019 was implemented to ensure adequate representation among variables of interest. The 2008 World Health Organization International Classification of Diseases for Oncology coding system, third revision (WHO ICD-O-3), was used to define pathology and set inclusion criteria for MACA.

Mucinous adenocarcinoma of the appendix diagnosis was defined as attribution of ICD-O-3 morphology codes 8480/3 ("mucinous adenocarcinoma"), 8481/3 ("mucin-producing adenocarcinoma"), or 8490/3 ("signet ring cell carcinoma") with corresponding appendiceal primary site (site code C18.2, "Appendix"). Cases of benign or *in situ* disease were excluded. Moreover, duplicate cases or cases of recurrent disease were excluded to ensure unique case data (Supplementary Fig. 1).

Three main cohorts were delineated to aid with analysis. Incidence statistics were derived from all unique patients with MACA diagnosis. Survival statistics were derived from all patients with an exclusive, first-time diagnosis of MACA with an age limitation of 20 to 79 years. Finally, a subgroup of patients with CRS-HIPEC rationale was defined to explore treatment strategy for MACA: this subgroup consisted of patients with regional or distant metastatic disease undergoing a surgical procedure, with or without intraoperative chemotherapy (see *Endpoints and Study Variables*). Patients with localized disease that could be treated with a simple appendectomy were excluded from this subgroup, as well as patients undergoing exclusive nonoperative management of metastatic MACA (Supplementary Fig. 1).

### Endpoints and Study Variables

The primary and secondary endpoints of this study were DSS and OS. Disease-specific survival was defined using the SEER cause-specific death classification, which algorithmically links death events to specific cancer diagnoses via sequence-based incorporation of perimortem risk factors.^23,24^ Patients with confirmed alive/dead status and known cause of death (if applicable) were included in the DSS analysis, whereas only confirmed alive/dead status was required for inclusion in the OS analysis. Incidence over time was calculated as a tertiary endpoint and was derived from raw annual rates of disease (see *Statistical Analysis and Ethics*).

Variables spanning epidemiology, demographics, and presentation at diagnosis of MACA were selected based on data completeness and relevance to the current analysis. Pretreatment factors included age, sex, race/ethnicity, year of diagnosis, geographic region, urban/rural status, median household income, histopathologic grade, lymph node status, and combined summary stage. Age was cohort-based and divided into either 5- or 10-year intervals, whereas race/ethnicity was based on the most recent five-category SEER-based race recode (April 2022). Geographic region, urban/rural status, and median household income were derived from standard U.S. Census classifications. Histopathologic grade was based on SEER composites for carcinoma grading simplified to a three-tier system ("well-differentiated - grade 1"; "moderately differentiated - grade 2"; "poorly differentiated - grade 3"), with all signet ring cell carcinomas automatically classified as grade 3 tumors.^21,25,26^ Similarly, tumors were categorized as "localized," "regional," or "distant" disease based on appendix-specific definitions outlined in the SEER Summary Stage Coding Manual (v3.1, 2018 revision), with these definitions being confinement to the primary organ, penetration into surrounding tissues and/or lymphatic spread to nearby structures (e.g., the mesoappendix or cecum), and metastasis to other intraperitoneal organs, respectively. For variables that were deprecated during the study period, composites were created to bridge results between original and successor variables, with preference given to SEER-defined composites or newer variables in cases of overlap.

Variables for postdiagnosis interventions included time to treatment and treatment strategy. For the latter, SEER sequence variables for operative/nonoperative interventions were used to define four broad categories of intervention: surgery only, surgery with other neoadjuvant/adjuvant systemic therapy, CRS-HIPEC only, and CRS-HIPEC with other neoadjuvant/adjuvant systemic therapy. Given the substantial differences in standard-of-care surgical management between localized and regional/metastatic MACA, these variables were only included in analyses for patients with CRS-HIPEC rationale.

### Statistical Analysis and Ethics

All data were accessed via SEER*Stat software (v8.4.1, National Cancer Institute, Bethesda, MD, March 29, 2023). Categorical variables were described as frequencies or percentages and were compared via χ^2^ test. Incidence trends were summarized as annual percent change (APC) and average annual percent change (AAPC) statistics with 95% confidence intervals (95% CI), calculated by using SEER Joinpoint Trend Analysis Software (v5.0.2, National Cancer Institute, May 25, 2023). All joinpoint models used empiric annual counts as a basis, weighted Bayesian information criterion (WBIC) for model selection, ordinary least squares (OLS) with assumed constant variance (homoscedasticity) for fit/standard error calculations, and empirical quantile method for 95% CI derivations. Survival was described via Kaplan-Meier analysis, with medians and log-rank *p* reported for comparison between groups. Multivariable Cox proportional hazards modeling was used to determine factors affecting survival with mortality risk quantified via hazard ratios (HR). In all analyses, *p*-values were two-sided with a statistical significance evaluated at the 0.05 alpha level; likewise, a 95% CI exclusive of 0 in joinpoint analysis or 1 in other analyses was considered to be statistically significant. All nonspecialized statistical calculations were performed using the R statistical programming language (v4.3.1, R Foundation for Statistical Computing, Vienna, Austria, June 16, 2023). Because all included data originated from a de-identified, publicly available database, institutional review board approval was waived for this analysis.

## Results

### Demographics, Pretreatment Factors, and Epidemiology

**Fig. 1:**
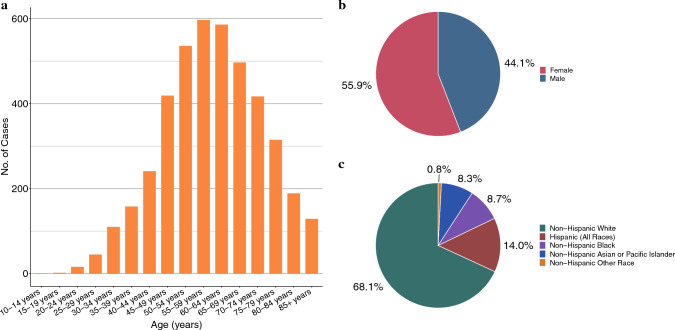
Patient demographic distributions, incidence cohort, categorized by (**a**) age, (**b**) sex, (**c**) race/ethnicity

In total, 4,258 unique patients with MACA were included in the analysis. The most common age of diagnosis was 50 to 69 years (52.0%), patients were majority female (55.9%), and most patients had non-Hispanic White background (68.1%, Fig. 1). Cases primarily originated from urban areas in the U.S. Northeast, South, and West, with relatively few cases occurring in the Midwest (3.5%) or in rural areas (4.3%, Supplementary Table 1). Distribution across tumor grades was fairly even (36.6% grade 1, 33.1% grade 2, and 30.3% grade 3), and most patients presented without evidence of lymphatic spread (71.1%); within this group, grade 1/2 disease represented the majority of diagnoses (80.2%). Despite this, most patients in the overall cohort presented with regional invasion or distant metastasis at the time of diagnosis (83.5%).

**Fig. 2:**
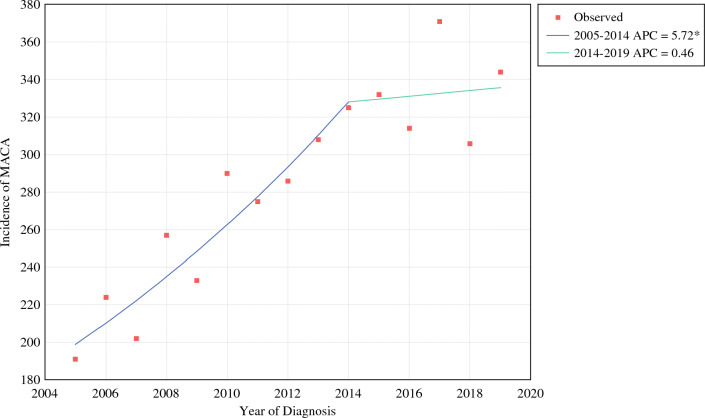
Incidence of mucinous adenocarcinoma of the appendix (MACA) with joinpoint regression curve. *Statistically significant difference from 0% APC

Incidence of MACA steadily increased between 2005 and 2014, with a leveling off period between 2015 and 2019 (Fig. 2). These findings correlated with a single-joinpoint regression trend (APC_1_ 5.72, APC_2_ 0.46; 2014 joinpoint) and an overall AAPC of 3.8 (95% CI 1.9–5.9). Using this as a baseline, similar analysis across demographic factors revealed comparatively higher AAPCs among females (4.4, 95% CI 3.2–5.7) and racial/ethnic minority patients, with apparent bimodal increased rates at age extremes (Table 1). Accounting for sample size (≥ 5% total count per variable), relative increases in AAPCs were likewise found in U.S. Northeast and South regions (4.8 and 5.4, respectively), as well as in medium and large urban centers (4.0 and 4.0, respectively), with mixed effects seen in high- and low-income household groups. Notably, higher AAPCs were found among grade 1 tumors (5.7, 95% CI 3.2–8.4), grade 2 tumors (4.2, 95% CI 1.8–6.7), and distant metastatic disease (6.2, 95% CI 2.3–10.0), reflecting a disproportionate increase in incidence for these groups.

**Table 1:** Total counts and annual average percent change in incidence by cohort in patients with mucinous adenocarcinoma of the appendix (MACA)

Variable	*n (%)*	Joinpoint(s)	2004-2019 AAPC (95% CI)
All	4258 (100.0%)	1	**3.8 (1.9-5.9)**
Age range, by decade (yr)			
10–19 ^†^	3 (0.1%)	1	**−5.4 (−9.0, -2.4)**
20–29	61 (1.4%)	0	**10.7 (0.7, 21.6)**
30–39	268 (6.3%)	0	**5.2 (2.1, 8.5)**
40–49	660 (15.5%)	1	−1.4 (−3.2, 0.6)
50–59	1133 (26.6%)	0	**2.4 (0.3, 4.6)**
60–69	1083 (25.4%)	1	**5.2 (3.2, 8.1)**
70–79	732 (17.2%)	0	**6.0 (3.0, 9.2)**
80+	318 (7.5%)	0	**4.2 (2.3-6.3)**
Sex			
Female	2380 (55.9%)	0	**4.4 (3.2, 5.7)**
Male	1878 (44.1%)	0	**3.7 (1.8, 5.8)**
Race/ethnicity			
Non-Hispanic White	2901 (68.1%)	1	**3.6 (2.1, 5.2)**
Hispanic (all races)	596 (14.0%)	0	**6.1 (3.3, 9.1)**
Non-Hispanic Black	370 (8.7%)	0	**5.9 (3.0, 9.0)**
Non-Hispanic Asian or Pacific Islander	355 (8.3%)	0	**4.8 (2.3, 7.5)**
Non-Hispanic other race^†^	36 (0.8%)	0	**14.2 (1.5, 28.3)**
U.S. Census region			
Northeast	819 (19.2%)	1	**4.8 (2.2, 7.1)**
South	956 (22.5%)	0	**5.4 (3.8, 7.0)**
Midwest	150 (3.5%)	1	2.4 (-5.8, 11.8)
West	2333 (54.8%)	1	**3.2 (1.2, 5.5)**
Urban/rural classification			
Urban (pop. >1,000,000)	2617 (61.5%)	0	**4.0 (2.2, 5.8)**
Urban (pop. 250,000–1,000,000)	882 (20.7%)	0	**4.0 (1.4, 6.8)**
Urban (pop. <250,000)	319 (7.5%)	0	3.2 (−0.2, 7.0)
Rural (urban-adjacent)	252 (5.9%)	0	3.5 (−0.9, 8.0)
Rural	185 (4.3%)	1	**6.5 (3.2, 10.0)**
Median household income			
More than $75,000	1431 (33.6%)	0	**5.2 (3.0, 7.5)**
$65,000–$74,999	1097 (25.8%)	0	2.5 (−2.8, 7.9)
$55,000–$64,999	875 (20.5%)	2	1.5 (−2.4, 8.1)
$45,000–$54,999	537 (12.6%)	0	**5.1 (1.5, 8.8)**
$35,000–$44,999	254 (6.0%)	1	**11.0 (3.6, 20.1)**
Less than $35,000	64 (1.5%)	0	**8.0 (2.7, 13.9)**
Histopathologic grade			
Well-differentiated - Grade 1	1260 (36.6%)	0	**5.7 (3.2, 8.4)**
Moderately differentiated - Grade 2	1142 (33.1%)	1	**4.2 (1.8, 6.7)**
Poorly differentiated - Grade 3	1044 (30.3%)	1	0.9 (−0.6, 2.3)
Lymph node status			
Negative	1863 (71.1%)	1	**3.6 (2.2, 5.4)**
Positive	756 (28.9%)	1	−0.4 (−4.0, 5.0)
Combined summary stage			
Localized	683 (16.5%)	0	0.5 (−1.2, 2.2)
Regional	980 (23.6%)	0	**3.5 (1.8, 5.2)**
Distant	2481 (59.9%)	1	**6.2 (2.3, 10.0)**

AAPC, average annual percent change

Bold indicates statistically significant difference from 0% AAPC

^†^Rate of 0.5 assigned to years with zero incidence to ensure model convergence

### Disease-Specific Survival, Overall Survival, and Prognostic Factors

A subset of 3,222 patients met age and exclusive-diagnosis criteria for survival analysis. Within this cohort, the median DSS was 118 (95% CI 103–138) months, and the median OS was 88 (95% CI 79–102) months. Significant survival differences were noted in different age groups, with particularly low median DSS observed in patients aged 20–29 years (78 vs. 102–118 months; *p* < 0.001; Supplementary Table 2). Similarly, patients aged 20–29 years were observed to have lower OS than other age groups except for those aged 70–79 years (78 vs. 53 vs. 88–107 months; *p* < 0.001). Geographic region and socioeconomic status were likewise significantly correlated with survival, with longest median survival rates noted for patients in the U.S. Northeast, in urban centers, and with high median household income. Classical markers of advanced disease, such as poorly differentiated tumors, positive lymph node status, and distant metastasis were associated with the lowest median survival rates among all factors measured (DSS 32, 31, and 53 months, respectively).

**Fig. 3:**
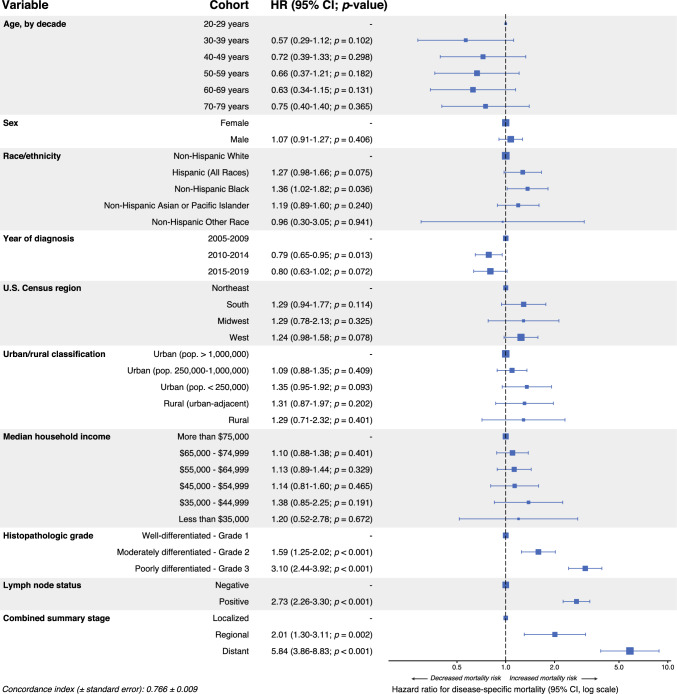
Multivariable Cox proportional hazards model for disease-specific survival (DSS), survival cohort

In DSS-based multivariable Cox regression, progressive stage was strongly associated with worse prognosis (distant metastasis HR 5.84, 95% CI 3.86–8.83; *p* < 0.001; Fig. 3). Positive lymph node status (HR 2.73, 95% CI 2.26–3.30; *p* < 0.001) and poorly differentiated disease (HR 3.10, 95% CI 2.44–3.92; *p* < 0.001) were similarly associated with worse outcomes. Notably, non-Hispanic Black race (HR 1.36, 95% CI 1.02–1.82; *p* = 0.036) also was independently associated with decreased DSS, whereas later year of diagnosis was associated with improved DSS (2010–2014 HR 0.79, 95% CI 0.65–0.95; *p* = 0.013). Parallel OS-based multivariable Cox regression modeling yielded similar results, with an additional association of Hispanic ethnicity with worse prognosis (HR 1.28, 95% CI 1.00–1.64; *p* = 0.048; Supplementary Fig. 2).

### Treatment Strategy and Survival in Patients with CRS-HIPEC Rationale

Within the survival cohort, 2,387 (74.1%) patients fit CRS-HIPEC rationale, although a minority ultimately underwent CRS-HIPEC (14.8; Table 2). Delineating by surgery/systemic therapy combination, 36.9% underwent surgery only, 48.3% underwent surgery with other nonintraoperative systemic therapy, 9.1% underwent CRS-HIPEC only, and 5.7% underwent CRS-HIPEC with other neoadjuvant/adjuvant systemic therapy. Broadly, a higher proportion of patients undergoing CRS-HIPEC procedures were male compared to those undergoing non-HIPEC surgeries (47.2% vs. 39.3%; *p* = 0.005), patients aged 70–79 years more commonly followed surgery-only treatment pathways compared to other age cohorts (21.5% vs. 10.9–14.3%; *p* < 0.001), and patients undergoing CRS-HIPEC with adjunctive systemic therapy were more likely to be non-Hispanic White race/ethnicity compared to those undergoing other treatment strategies (74.5% vs. 61.9–67.7%; *p* = 0.007). Similarly, CRS-HIPEC occurred more frequently during later time periods (2015–2019, 49.7% vs. 38.9%; *p* < 0.001), in the U.S. West region (61.3% vs. 55.0%; *p* < 0.001), and in large urban centers (69.5% vs. 60.6%; *p* = 0.001).

**Table 2:** Comparison of patients with mucinous adenocarcinoma of the appendix (MACA) receiving cytoreductive surgery with hyperthermic intraperitoneal chemotherapy (CRS-HIPEC) versus other surgical interventions

Variable	Total (*n* = 2,387)	Surgery Without HIPEC (*n* = 2,033)	CRS-HIPEC (*n* = 354)	*p* value	Surgery only (*n* = 881)	Surgery and other systemic therapy (*n* = 1,152)	CRS-HIPEC only (*n* = 217)	CRS-HIPEC with systemic therapy (*n* = 137)	*p* value
Age range, by decade				0.446					**< 0.001**
20-29 years	40 (1.7%)	35 (1.7%)	5 (1.4%)		14 (1.6%)	21 (1.8%)	1 (0.5%)	4 (2.9%)	
30-39 years	181 (7.6%)	150 (7.4%)	31 (8.8%)		57 (6.5%)	93 (8.1%)	20 (9.2%)	11 (8.0%)	
40-49 years	461 (19.3%)	387 (19.0%)	74 (20.9%)		143 (16.2%)	244 (21.2%)	44 (20.3%)	30 (21.9%)	
50-59 years	727 (30.5%)	611 (30.1%)	116 (32.8%)		256 (29.1%)	355 (30.8%)	72 (33.2%)	44 (32.1%)	
60-69 years	608 (25.5%)	526 (25.9%)	82 (23.2%)		222 (25.2%)	304 (26.4%)	49 (22.6%)	33 (24.1%)	
70-79 years	370 (15.5%)	324 (15.9%)	46 (13.0%)		189 (21.5%)	135 (11.7%)	31 (14.3%)	15 (10.9%)	
Sex				**0.005**					**0.048**
Female	1,421 (59.5%)	1,234 (60.7%)	187 (52.8%)		531 (60.3%)	703 (61.0%)	114 (52.5%)	73 (53.3%)	
Male	966 (40.5%)	799 (39.3%)	167 (47.2%)		350 (39.7%)	449 (39.0%)	103 (47.5%)	64 (46.7%)	
Race/ethnicity				0.211					**0.007**
Non-Hispanic White	1,568 (65.7%)	1,325 (65.2%)	243 (68.6%)		545 (61.9%)	780 (67.7%)	141 (65.0%)	102 (74.5%)	
Hispanic (all races)	367 (15.4%)	319 (15.7%)	48 (13.6%)		164 (18.6%)	155 (13.5%)	33 (15.2%)	15 (10.9%)	
Non-Hispanic Black	210 (8.8%)	182 (9.0%)	28 (7.9%)		70 (7.9%)	112 (9.7%)	21 (9.7%)	7 (5.1%)	
Non-Hispanic Asian or Pacific Islander	220 (9.2%)	185 (9.1%)	35 (9.9%)		89 (10.1%)	96 (8.3%)	22 (10.1%)	13 (9.5%)	
Non-Hispanic other race	22 (0.9%)	22 (1.1%)	0 (0.0%)		13 (1.5%)	9 (0.8%)	0 (0.0%)	0 (0.0%)	
Year of diagnosis				**< 0.001**					**< 0.001**
2005-2009	589 (24.7%)	544 (26.8%)	45 (12.7%)		333 (37.8%)	211 (18.3%)	28 (12.9%)	17 (12.4%)	
2010-2014	832 (34.9%)	699 (34.4%)	133 (37.6%)		249 (28.3%)	450 (39.1%)	88 (40.6%)	45 (32.8%)	
2015-2019	966 (40.5%)	790 (38.9%)	176 (49.7%)		299 (33.9%)	491 (42.6%)	101 (46.5%)	75 (54.7%)	
U.S. Census region				**< 0.001**					**< 0.001**
Northeast	444 (18.6%)	404 (19.9%)	40 (11.3%)		146 (16.6%)	258 (22.4%)	22 (10.1%)	18 (13.1%)	
South	529 (22.2%)	450 (22.1%)	79 (22.3%)		169 (19.2%)	281 (24.4%)	39 (18.0%)	40 (29.2%)	
Midwest	79 (3.3%)	61 (3.0%)	18 (5.1%)		24 (2.7%)	37 (3.2%)	15 (6.9%)	3 (2.2%)	
West	1,335 (55.9%)	1,118 (55.0%)	217 (61.3%)		542 (61.5%)	576 (50.0%)	141 (65.0%)	76 (55.5%)	
Urban/rural classification				**0.001**					**0.015**
Urban (pop. > 1,000,000)	1,477 (61.9%)	1,231 (60.6%)	246 (69.5%)		516 (58.6%)	715 (62.1%)	147 (67.7%)	99 (72.3%)	
Urban (pop. 250,000-1,000,000)	499 (20.9%)	451 (22.2%)	48 (13.6%)		211 (24.0%)	240 (20.9%)	29 (13.4%)	19 (13.9%)	
Urban (pop. < 250,000)	174 (7.3%)	142 (7.0%)	32 (9.0%)		67 (7.6%)	75 (6.5%)	22 (10.1%)	10 (7.3%)	
Rural (urban-adjacent)	138 (5.8%)	121 (6.0%)	17 (4.8%)		47 (5.3%)	74 (6.4%)	11 (5.1%)	6 (4.4%)	
Rural	97 (4.1%)	86 (4.2%)	11 (3.1%)		39 (4.4%)	47 (4.1%)	8 (3.7%)	3 (2.2%)	
Median household income				0.067					0.156
More than $75,000	816 (34.2%)	707 (34.8%)	109 (30.8%)		288 (32.7%)	419 (36.4%)	70 (32.3%)	39 (28.5%)	
$65,000 - $74,999	608 (25.5%)	523 (25.7%)	85 (24.0%)		244 (27.7%)	279 (24.2%)	54 (24.9%)	31 (22.6%)	
$55,000 - $64,999	495 (20.7%)	400 (19.7%)	95 (26.8%)		173 (19.6%)	227 (19.7%)	52 (24.0%)	43 (31.4%)	
$45,000 - $54,999	293 (12.3%)	250 (12.3%)	43 (12.1%)		115 (13.1%)	135 (11.7%)	27 (12.4%)	16 (11.7%)	
$35,000 - $44,999	137 (5.7%)	121 (6.0%)	16 (4.5%)		46 (5.2%)	75 (6.5%)	11 (5.1%)	5 (3.6%)	
Less than $35,000	38 (1.6%)	32 (1.6%)	6 (1.7%)		15 (1.7%)	17 (1.5%)	3 (1.4%)	3 (2.2%)	
Histopathologic grade				**< 0.001**					**< 0.001**
Well-differentiated - Grade 1	726 (35.4%)	591 (33.6%)	135 (45.9%)		314 (41.4%)	277 (27.7%)	95 (52.2%)	40 (35.7%)	
Moderately differentiated - Grade 2	690 (33.6%)	577 (32.8%)	113 (38.4%)		263 (34.7%)	314 (31.4%)	67 (36.8%)	46 (41.1%)	
Poorly differentiated - Grade 3	635 (31.0%)	589 (33.5%)	46 (15.6%)		181 (23.9%)	408 (40.8%)	20 (11.0%)	26 (23.2%)	
Lymph node status				**< 0.001**					**< 0.001**
Negative	1,070 (64.9%)	868 (62.0%)	202 (81.8%)		414 (72.6%)	454 (54.6%)	131 (87.3%)	71 (73.2%)	
Positive	578 (35.1%)	533 (38.0%)	45 (18.2%)		156 (27.4%)	377 (45.4%)	19 (12.7%)	26 (26.8%)	
Combined summary stage				**< 0.001**					**< 0.001**
Regional	678 (28.4%)	653 (32.1%)	25 (7.1%)		337 (38.3%)	316 (27.4%)	17 (7.8%)	8 (5.8%)	
Distant	1,709 (71.6%)	1,380 (67.9%)	329 (92.9%)		544 (61.7%)	836 (72.6%)	200 (92.2%)	129 (94.2%)	
Time to treatment				**< 0.001**					**< 0.001**
Less than 1 month	1,783 (74.9%)	1,600 (78.8%)	183 (52.0%)		741 (84.3%)	859 (74.6%)	110 (51.2%)	73 (53.3%)	
1-2 months	342 (14.4%)	256 (12.6%)	86 (24.4%)		73 (8.3%)	183 (15.9%)	41 (19.1%)	45 (32.8%)	
2-3 months	137 (5.8%)	104 (5.1%)	33 (9.4%)		27 (3.1%)	77 (6.7%)	25 (11.6%)	8 (5.8%)	
More than 3 months	120 (5.0%)	70 (3.4%)	50 (14.2%)		38 (4.3%)	32 (2.8%)	39 (18.1%)	11 (8.0%)	
Disease-specific survival				**< 0.001**					**< 0.001**
Median survival, months (95% CI)	97 (80, 114)	81 (75, 100)	129 (106, NA)		126 (98, NA)	57 (50, 76)	NA (NA, NA)	104 (53, NA)	
Number of events	909	817	92		318	499	49	43	
Overall survival				**< 0.001**					**< 0.001**
Median survival, months (95% CI)	78 (69, 87)	69 (60, 79)	109 (104, NA)		89 (76, 117)	53 (48, 65)	116 (106, NA)	104 (53, NA)	
Number of events	1023	920	103		379	541	56	47	

CRS-HIPEC, cytoreductive surgery and hyperthermic intraperitoneal chemotherapy

Bold indicates statistically significant difference from 0% AAPC

Clinically, CRS-HIPEC was more frequently performed on grade 1 tumors (45.9% vs. 33.6%; *p* < 0.001), without neoadjuvant/adjuvant systemic therapy (52.2% vs. 27.7–41.4%; *p* < 0.001; Table 2). Likewise, CRS-HIPEC was less frequently performed on patients with positive lymph nodes (18.2% vs. 38.0%; *p* < 0.001) and was more frequently performed on patients with distant metastasis (92.9% vs. 67.9%; *p* < 0.001). Management with CRS-HIPEC generally involved longer times to treatment than non-HIPEC regimens (> 3 months 14.2% vs. 3.4%; *p* < 0.001).

Patients who underwent CRS-HIPEC had increased median survival compared to those undergoing non-HIPEC surgeries (DSS 129 vs. 81 months, OS 109 vs. 69 months; *p* < 0.001; Supplementary Fig. 3). Moreover, patients who underwent CRS-HIPEC only had the longest median survival among treatment subgroups (OS 116 vs. 53–104 months; *p* < 0.001). Survival curves for various other factors within the CRS-HIPEC rationale cohort revealed worse prognosis but similar trends to the survival cohort (Supplementary Table 3).

**Fig. 4:**
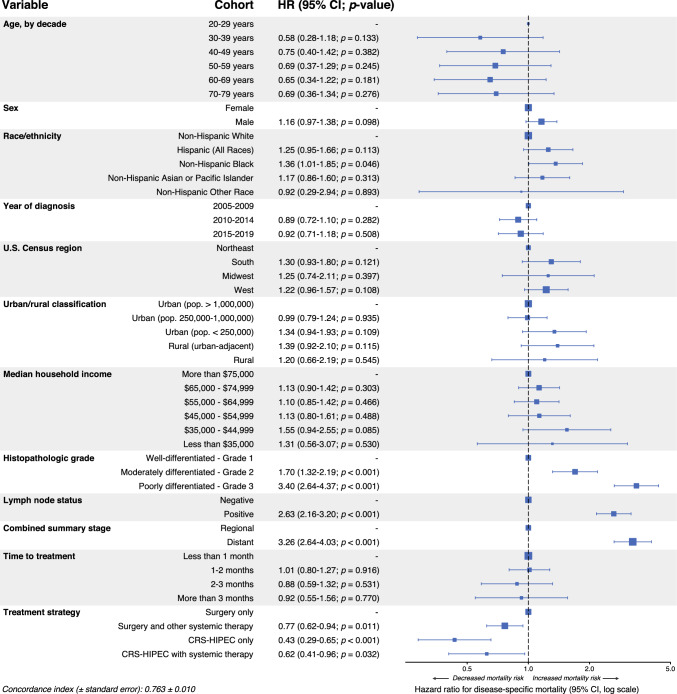
Multivariable Cox proportional hazards model for disease-specific survival (DSS), cytoreductive surgery with hyperthermic intraperitoneal chemotherapy (CRS-HIPEC) rationale cohort

In DSS-based multivariable Cox proportional hazards modeling, CRS-HIPEC-only therapy was strongly associated with increased DSS within the CRS-HIPEC rationale subcohort (HR 0.43, 95% CI 0.29–0.65; *p* < 0.001), followed by CRS-HIPEC with systemic therapy (HR 0.62, 95% CI 0.41–0.96; *p* = 0.032) and surgery with other systemic therapy (HR 0.77, 95% CI 0.62-0.94; *p* = 0.011; Fig. 4). Other conventional factors correlated with worse prognosis, such as grade 2 disease (HR 1.70, 95% CI 1.32–2.19; *p* < 0.001), grade 3 disease (HR 3.40, 95% CI 2.64–4.37; *p* < 0.001), positive lymph node status (HR 2.63, 95% CI 2.16–3.20; *p* < 0.001), and distant metastasis (HR 3.26, 95% CI 2.64–4.03; *p* < 0.001). Demographically, only non-Hispanic Black race was significantly associated with worse DSS (HR 1.36, 95% CI 1.01–1.85; *p* = 0.046). Parallel OS analysis revealed similar protective trends with treatment, with male sex independently associated with worse survival (HR 1.24, 95% CI 1.05–1.46; *p* = 0.010) and non-Hispanic Black race no longer significantly associated with worse outcomes (Supplementary Fig. 4).

**Fig. 5:**
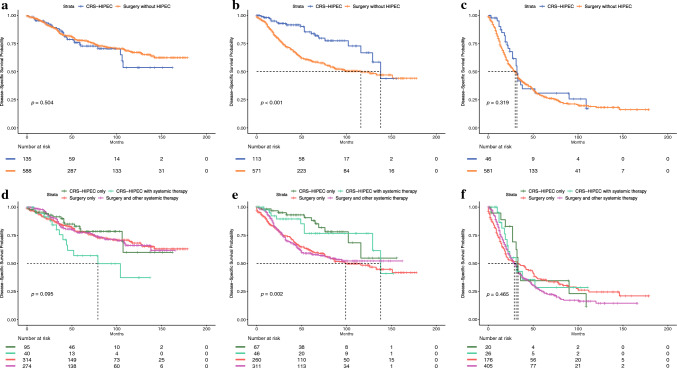
Kaplan-Meier disease-specific survival (DSS) curves, cytoreductive surgery with hyperthermic intraperitoneal chemotherapy (CRS-HIPEC) rationale cohort, by grade and treatment strategy: (**a**) CRS-HIPEC vs. other surgery, grade 1 disease; (**b**) CRS-HIPEC vs. other surgery, grade 2 disease; (**c**) CRS-HIPEC vs. other surgery, grade 3 disease; (**d**) CRS-HIPEC vs. other surgery by treatment type, grade 1 disease; (**e**) CRS-HIPEC vs. other surgery by treatment type, grade 2 disease; (**f**) CRS-HIPEC vs. other surgery by treatment type, grade 3 disease

Because histopathologic grade had the strongest association with worse DSS and OS in the CRS-HIPEC rationale cohort, in-depth examination of survival curves based on tumor grade and treatment strategy was performed (Fig. 5 and Supplementary Fig. 5). For both DSS and OS, these curves revealed significantly improved survival with CRS-HIPEC therapy for grade 2 disease exclusively (median DSS 138 vs. 116 months, median OS 138 vs. 87 months; *p* < 0.001). Similarly, when categorized by surgery/systemic therapy combination, CRS-HIPEC-only and CRS-HIPEC with systemic therapy treatment regimens correlated with prolonged DSS/OS in grade 2 disease compared with surgery alone or surgery with systemic therapy. By contrast, treatment with CRS-HIPEC showed no significant improvement in survival in grade 1 or grade 3 disease regardless of categorization, and CRS-HIPEC with systemic therapy was in fact associated with worse DSS/OS in grade 1 tumors compared with other modalities, although this correlation was not significant (median DSS 79 months; *p* = 0.095).

## Discussion

By using population-level data to describe the demographics, socioeconomic status, clinical presentation, and treatment regimens of patients with MACA, this study identifies and compares several novel factors that may significantly influence outcomes in this disease. Along with classical markers of disease progression, both sex and racial/ethnic background were found in this analysis to be independent predictors of DSS and OS for patients with MACA, with additional factors such as year of diagnosis further affecting MACA-related mortality. Likewise, treatment methodology was also found to significantly impact outcomes, with CRS-HIPEC-based treatment associated with improved survival among patients with moderately differentiated disease in particular. Given the increasing incidence of MACA not only in low-grade/metastatic disease cohorts but also among racial/ethnic minority populations, these results may help to refine treatment strategy with the goal of optimizing long-term survival.

Disease grade is a well-established prognostic indicator for MACA, with grade 2 and 3 disease shown to have a 1.3- to 2.7-fold and 2.7- to 5.6-fold increased hazard for overall mortality, respectively.^4,20,27,28^ By contrast, CRS-HIPEC has been demonstrated to improve MACA-related survival, with multiple phase II studies demonstrating decreased mortality, decreased recurrence, longer progression-free survival, and fewer reoperations compared to conventional therapies.^6,9,10,18,29,30^ This proven efficacy has led to the frequent recommendation of CRS-HIPEC for treatment of distant intraperitoneal metastasis from MACA (i.e., PMP), with the procedure currently utilized to treat most patients with low-grade disease, many patients with moderate-grade disease, and highly selected cohorts with high-grade disease.^3,9,10,21^ By stratifying treatment strategy by tumor grade, this current analysis qualifies this recommendation by suggesting that the bulk of CRS-HIPEC response in MACA originates from grade 2 tumors, despite fewer than half of documented procedures (38.4%) being performed on these patients. Moreover, the relatively small impact of CRS-HIPEC-based therapy on grade 1 DSS/OS seen in this analysis suggests that the additional benefit of HIPEC beyond a complete or optimal CRS is unclear, and CRS-only therapies may be viable to treat limited, low-grade disease while avoiding HIPEC-associated morbidity. As incidence rates of grade 1, grade 2, and metastatic MACA have been asymmetrically increasing in recent years (AAPC 5.7, 4.2, and 6.2, respectively), tumor grade should therefore comprise an integral part of preoperative planning for CRS-HIPEC in patients with MACA, with acknowledgment that CRS-HIPEC may not ultimately extend long-term survival in patients with grade 1 or grade 3 tumors.

Despite the utility of CRS-HIPEC in treating MACA, the role of adjunct systemic therapy remains controversial. Several early studies have suggested some therapeutic benefit with neoadjuvant chemotherapy, with one notable prospective analysis of 34 patients reporting a 29% complete or near-complete histopathologic response rate among high-grade MACA patients treated with FOLFOX chemotherapy before CRS-HIPEC.^31^ More recent analyses, however, have demonstrated no clear survival benefit in MACA with neoadjuvant chemotherapy,^15–17,32,33^ and several have shown worse survival among patients receiving neoadjuvant chemotherapy compared with CRS-HIPEC alone.^6,12,30^ By comparison, adjuvant systemic therapy has shown mixed outcomes for MACA, with one large database analysis demonstrating OS benefit among patients receiving adjuvant chemotherapy with stage IV MACA, but only among those with moderately or poorly differentiated disease.^4^ Conversely, several smaller studies have suggested that adjuvant systemic therapy has either an equivocal^17,34^ or negative effect on OS.^12^ Given this variance in data, general practice guidelines for MACA typically reserve adjunct therapy for cases of high-grade disease only, with CRS-HIPEC monotherapy recommended for well-differentiated tumors.^3,5,9,21^ Accordingly, while adjunct systemic therapy in this current analysis correlated with improved DSS and OS compared with surgery alone, its effect was eclipsed by primary CRS-HIPEC management and fluctuated depending on disease grade, with combined CRS-HIPEC and systemic therapy corresponding with worse DSS in grade 1 disease.

Because of its rarity, few studies have analyzed demographic and socioeconomic trends with MACA. In general, MACA most commonly occurs in the sixth and seventh decades of life with a slight female preponderance (50–57%), which corresponds with this current analysis.^1,4,18,20,22,35^ Similarly, several studies report a prevalence of MACA among individuals with White racial/ethnic background (86–89%),^1,20,22^ which is likely observed at a lower level in this current study (68.1%) due to the recent reclassification of Hispanic ethnicity in SEER demographic variables.^36^ Importantly, despite comprising only a fraction of total caseload in this study, patients with Hispanic (14.0%) or non-Hispanic Black background (8.7%) were observed to have nearly twice the increase in incidence compared to non-Hispanic White patients (AAPC 5.9/6.1 vs. 3.6), marking a fundamental demographic shift in MACA disease burden. Moreover, both groups were independently associated with worse survival in this analysis (OS and DSS/OS, respectively), highlighting an increased need to address and improve MACA treatment strategies in these populations. Outside of this correlation, male sex was found to be associated with worse OS within the CRS-HIPEC rationale subgroup analysis, suggesting that other demographic factors may further influence survival in patients with MACA.^1,3,5^

Despite being empirically associated with DSS and/or OS in this current analysis, factors such as age, urban/rural classification, and median household income were not significantly associated with survival in subsequent multivariable models. These results underscore the complex nature of MACA and the necessity of using adjusted models to predict outcomes in this disease.^3,28,33^ In particular, younger patients aged 20 to 29 years were found in this analysis to paradoxically have the lowest and second-lowest median DSS and OS respectively while simultaneously having a higher proportion of positive prognostic factors, such as grade 1/2 tumors (81.6% vs. 66.2–78.5%, *p* = 0.047). In this case, this population was also found to have a higher proportion of individuals belonging to demographic groups associated with worse OS, such as Hispanic ethnicity (25.5% vs. 12.1–23.8%; *p* < 0.001) and non-Hispanic Black race (10.9% vs. 7.3–13.3%, *p* < 0.001), although these findings likely do not fully explain the trend toward worse survival in this cohort.^3,10^

By design, this analysis uses a national database, which allows for broad tracking of incidence rates and survival factors but lacks the granularity offered by smaller cohort analyses. As such, this study is limited in that it does not account for numerous clinical factors proven to influence MACA-related outcomes, such as baseline functional status, degree of tumor invasion, peritoneal cancer index (PCI), metastatic tumor location, specific resections/anastomoses performed during CRS-HIPEC, completeness of cytoreduction (CC score or R score), chemotherapeutic agent(s) used with dosing information, and reoperations and/or serial debulking procedures.^3,5,7–11,13,20^ In particular, completeness of cytoreduction has historically been strongly linked to the success or failure of CRS-HIPEC therapy, with the current literary consensus suggesting that CRS-HIPEC only extends survival in MACA if all macroscopic disease ≥ 2.5 mm is removed during surgery (i.e., CC-0/CC-1 or R1/R2a cytoreduction).^6,19,37–42^ While metrics measuring CRS-HIPEC quality were not available for this current analysis, this difference in MACA-related survival based on CRS-HIPEC quality has been quantified in various other studies. For example, in one of the first randomized controlled trials analyzing the effect of CRS-HIPEC on MACA, complete cytoreduction was found to be the most important prognostic indicator of long-term DSS, with R1 cytoreduction corresponding with a median DSS of 48 months versus fewer than 18 months in R2a/R2b cytoreductions.^19^ Similarly, in a large, multi-institutional, retrospective review of 2,259 patients with appendiceal neoplasm-related PMP, CC-0/CC-1 cytoreduction was the strongest clinical predictor of OS among all measured variables, with 5-year OS rates of 85–91% versus 33% in low-grade tumors and 68–72% versus 0% in high-grade tumors.^6^ By extension, other clinical factors such as functional status may also indirectly affect outcomes, not only by estimating patient fitness for therapy but also by influencing the likelihood of pursuing aggressive treatment strategies, such as CRS-HIPEC: in several studies, high Eastern Cooperative Oncology Group (ECOG) scores have been linked with both worse OS^40,41^ and increased rates of conservative disease management without CRS-HIPEC.^3,5,10^ Therefore, while this study establishes a general relationship between tumor grade and CRS-HIPEC efficacy, further research is needed to incorporate more detailed clinicopathologic factors in the broader context of tumor biology, which will allow for more accurate prediction of MACA-related survival.

This study has several other limitations. Regarding outcomes, this study focuses exclusively on incidence and survival, which does not address alternative outcomes such as symptom palliation and quality of life. Given the insidious and obstructive nature of MACA, these outcomes are highly relevant and are often the goal of interventions rather than long-term survival.^3,10,15,30^ This study also analyzed MACA only, excluding other appendiceal tumors that also produce PMP, such as low- and high-grade appendiceal mucinous neoplasms (LAMNs/HAMNs), as well as disseminated peritoneal adenomucosis (DPAM); this decision was made to mitigate ambiguity when discussing potentially overlapping pathologies (e.g., well-differentiated MACA and LAMN) while ensuring consistent data quality throughout the study period.^3,21,43,44^ For a similar reason, AJCC 8th edition guidelines were used due to their more general categorization of MACA tumors and greater compatibility with historic ICD-O-3 coding structure, at the expense of some additional tumor subclassifications (e.g., SCC).^3,26^ Technical data limitations were also present: while the SEER database boasts a broad catchment area with registries representing approximately 48% of the U.S. population, its coverage is not comprehensive and may miss patients treated with CRS-HIPEC at nonaffiliated centers.^45^ Last, certain composite variables, such as treatment strategy, were created by merging several empiric variables, causing loss of data resolution; in such cases, this was performed to simplify results and ensure model convergence in downstream analysis.

## Conclusions

Mucinous adenocarcinoma of the appendix is a complex disease with increasing incidence and variable survival. Classical indicators of aggressive or advanced disease such as high tumor grade, positive lymph node status, and distant metastasis have a strong negative impact on both DSS and OS. Other demographic factors, such as Hispanic/Black background and male sex, are independently associated with worse survival. Among patients with CRS-HIPEC rationale, CRS-HIPEC confers a significant survival benefit, although this benefit is limited to moderately differentiated disease. Further analysis is needed to enhance risk stratification of MACA, especially because low-grade and metastatic tumors now form the majority of newly diagnosed disease, and incidence rates among racial/ethnic minority patients have been disproportionately rising.

### Supplementary Information

Below is the link to the electronic supplementary material.

Supplementary file1 Supplementary Table 1 Incidence of demographic attributes and presenting characteristics in patients with mucinous adenocarcinoma of the appendix (MACA) (17 KB)

Supplementary file2 Supplementary Table 2 Comparison of median disease-specific survival and overall survival for patients with mucinous adenocarcinoma of the appendix (MACA) (10 KB)

Supplementary file3 Supplementary Table 3 Comparison of median disease-specific survival and overall survival for patients with mucinous adenocarcinoma of the appendix (MACA) and cytoreductive surgery with hyperthermic intraperitoneal chemotherapy (CRS-HIPEC) rationale (11 KB)

Supplementary file4 Supplementary Fig. 1 Flow diagram of cohort selection, with inclusion and exclusion criteria (62 KB)

Supplementary file5 Supplementary Fig. 2 Multivariable Cox proportional hazards model for overall survival (OS), survival cohort (90 KB)

Supplementary file6 Supplementary Fig. 3 Kaplan-Meier survival curves, cytoreductive surgery with hyperthermic intraperitoneal chemotherapy (CRS-HIPEC) rationale cohort: (a) disease-specific survival (DSS) of CRS-HIPEC vs. other surgery; (b) DSS of CRS-HIPEC vs. other surgery, by surgery/systemic therapy combination; (c) overall survival (OS) of CRS-HIPEC vs. other surgery; (d) OS of CRS-HIPEC vs. other surgery, by surgery/systemic therapy combination (925 KB)

Supplementary file7 Supplementary Fig. 4 Multivariable Cox proportional hazards model for overall survival (OS), cytoreductive surgery with hyperthermic intraperitoneal chemotherapy (CRS-HIPEC) rationale cohort (98 KB)

Supplementary file8 Supplementary Fig. 5 Kaplan-Meier overall survival (OS) curves, cytoreductive surgery with hyperthermic intraperitoneal chemotherapy (CRS-HIPEC) rationale cohort, by grade and treatment strategy: (a) CRS-HIPEC vs. other surgery, grade 1 disease; (b) CRS-HIPEC vs. other surgery, grade 2 disease; (c) CRS-HIPEC vs. other surgery, grade 3 disease; (d) CRS-HIPEC vs. other surgery by treatment type, grade 1 disease; (e) CRS-HIPEC vs. other surgery by treatment type, grade 2 disease; (f) CRS-HIPEC vs. other surgery by treatment type, grade 3 disease (847 KB)

## References

[1] McCusker ME, Coté TR, Clegg LX, Sobin LH. Primary malignant neoplasms of the appendix: A population-based study from the surveillance, epidemiology and end-results program, 1973–1998. *Cancer*. 2002;94(12):3307–12. 10.1002/cncr.10589. 12115365 10.1002/cncr.10589

[2] Marmor S, Portschy PR, Tuttle TM, Virnig BA. The rise in appendiceal cancer incidence: 2000–2009. *J Gastrointest Surg*. 2015;19(4):743–50. 10.1007/s11605-014-2726-7. 25560182 10.1007/s11605-014-2726-7

[3] Govaerts K, Lurvink R, De Hingh I, et al. Appendiceal tumours and pseudomyxoma peritonei: Literature review with PSOGI/EURACAN clinical practice guidelines for diagnosis and treatment. *Eur J Surg Oncol*. 2021;47(1):11–35. 10.1016/j.ejso.2020.02.012. 32199769 10.1016/j.ejso.2020.02.012

[4] Asare EA, Compton CC, Hanna N, et al. The impact of stage, grade, and mucinous histology on the efficacy of systemic chemotherapy in adenocarcinomas of the appendix: Analysis of the National Cancer Data Base (NCDB). *Cancer*. 2016;122(2):213–21. 10.1002/cncr.29744. 26506400 10.1002/cncr.29744 PMC4860278

[5] Chicago Consensus Working Group. The Chicago Consensus on Peritoneal Surface Malignancies: Management of Appendiceal Neoplasms. *Ann Surg Oncol*. 2020;27(6):1753–60. 10.1245/s10434-020-08316-w. 32285275 10.1245/s10434-020-08316-w

[6] Chua TC, Moran BJ, Sugarbaker PH, et al. Early- and long-term outcome data of patients with pseudomyxoma peritonei from appendiceal origin treated by a strategy of cytoreductive surgery and hyperthermic intraperitoneal chemotherapy. *J Clin Oncol*. 2012;30(20):2449–56. 10.1200/JCO.2011.39.7166. 22614976 10.1200/JCO.2011.39.7166

[7] Guaglio M, Sinukumar S, Kusamura S, et al. Clinical surveillance after macroscopically complete surgery for low-grade appendiceal mucinous neoplasms (LAMN) with or without limited peritoneal spread: long-term results in a prospective series. *Ann Surg Oncol*. 2018;25(4):878–84. 10.1245/s10434-017-6305-5. 10.1245/s10434-017-6305-5 29270877

[8] Baratti D, Kusamura S, Milione M, Bruno F, Guaglio M, Deraco M. Validation of the recent PSOGI pathological classification of pseudomyxoma peritonei in a single-center series of 265 patients treated by cytoreductive surgery and hyperthermic intraperitoneal chemotherapy. *Ann Surg Oncol*. 2018;25(2):404–13. 10.1245/s10434-017-6252-1. 29159742 10.1245/s10434-017-6252-1

[9] Hoehn RS, Rieser CJ, Choudry MH, Melnitchouk N, Hechtman J, Bahary N. Current management of appendiceal neoplasms. *Am Soc Clin Oncol Educ Book*. 2021;41:118–32. 10.1200/EDBK_321009. 10.1200/EDBK_321009 33770459

[10] Choudry HA, Pai RK, Bartlett DL. Mucinous Appendiceal Tumors. In: Morita SY, Balch CM, Klimberg VS, Pawlik TM, Posner MC, Tanabe KK, eds. *Textbook of Complex General Surgical Oncology*. McGraw-Hill Education; 2018. https://accesssurgery.mhmedical.com/content.aspx?aid=1145763322

[11] Jacquet P, Sugarbaker PH. Clinical research methodologies in diagnosis and staging of patients with peritoneal carcinomatosis. *Cancer Treat Res*. 1996;82:359–74. 10.1007/978-1-4613-1247-5_23. 8849962 10.1007/978-1-4613-1247-5_23

[12] Cummins KA, Russell GB, Votanopoulos KI, Shen P, Stewart JH, Levine EA. Peritoneal dissemination from high-grade appendiceal cancer treated with cytoreductive surgery (CRS) and hyperthermic intraperitoneal chemotherapy (HIPEC). *J Gastrointest Oncol*. 2016;7(1):3–9. 10.3978/j.issn.2078-6891.2015.101. 26941979 10.3978/j.issn.2078-6891.2015.101 PMC4754307

[13] Sugarbaker PH, Chang D. Results of treatment of 385 patients with peritoneal surface spread of appendiceal malignancy. *Ann Surg Oncol*. 1999;6(8):727–31. 10.1007/s10434-999-0727-7. 10622499 10.1007/s10434-999-0727-7

[14] Munoz-Zuluaga C, Sardi A, King MC, et al. Outcomes in peritoneal dissemination from signet ring cell carcinoma of the appendix treated with cytoreductive surgery and hyperthermic intraperitoneal chemotherapy. *Ann surg oncol*. 2019;26(2):473–81. 10.1245/s10434-018-7007-3. 30523470 10.1245/s10434-018-7007-3

[15] Bijelic L, Kumar AS, Stuart OA, Sugarbaker PH. Systemic chemotherapy prior to cytoreductive surgery and HIPEC for carcinomatosis from appendix cancer: impact on perioperative outcomes and short-term survival. *Gastroenterol Res Pract*. 2012;2012:1–6. 10.1155/2012/163284. 10.1155/2012/163284 PMC3412098 22899903

[16] Milovanov V, Sardi A, Ledakis P, et al. Systemic chemotherapy (SC) before cytoreductive surgery and hyperthermic intraperitoneal chemotherapy (CRS/HIPEC) in patients with peritoneal mucinous carcinomatosis of appendiceal origin (PMCA). *Eur J Surg Oncol EJSO*. 2015;41(5):707–12. 10.1016/j.ejso.2015.01.005. 25633641 10.1016/j.ejso.2015.01.005

[17] Blackham AU, Swett K, Eng C, et al. Perioperative systemic chemotherapy for appendiceal mucinous carcinoma peritonei treated with cytoreductive surgery and hyperthermic intraperitoneal chemotherapy: perioperative chemo for appendiceal MCP. *J Surg Oncol*. 2014;109(7):740–5. 10.1002/jso.23547. 24375188 10.1002/jso.23547 PMC4010799

[18] Austin F, Mavanur A, Sathaiah M, et al. Aggressive Management of Peritoneal Carcinomatosis from Mucinous Appendiceal Neoplasms. *Ann surg oncol*. 2012;19(5):1386–93. 10.1245/s10434-012-2241-6. 22302270 10.1245/s10434-012-2241-6 PMC4100545

[19] Verwaal VJ, Bruin S, Boot H, Van Slooten G, Van Tinteren H. 8-Year follow-up of randomized trial: cytoreduction and hyperthermic intraperitoneal chemotherapy versus systemic chemotherapy in patients with peritoneal carcinomatosis of colorectal cancer. *Ann Surg Oncol*. 2008;15(9):2426–32. 10.1245/s10434-008-9966-2. 18521686 10.1245/s10434-008-9966-2

[20] Levinsky NC, Morris MC, Wima K, et al. Should we be doing cytoreductive surgery with HIPEC for signet ring cell appendiceal adenocarcinoma? A study from the US HIPEC Collaborative. *J Gastrointest Surg*. 2020;24(1):155–64. 10.1007/s11605-019-04336-4. 31428960 10.1007/s11605-019-04336-4

[21] Kang DW, Kim Bh, Kim JM, et al. Standardization of the pathologic diagnosis of appendiceal mucinous neoplasms. *J Pathol Transl Med*. 2021;55(4):247–64. 10.4132/jptm.2021.05.28. 34233112 10.4132/jptm.2021.05.28 PMC8353140

[22] Orchard P, Preece R, Thomas MG, et al. Demographic trends in the incidence of malignant appendiceal tumours in England between 1995 and 2016: Population-based analysis. *BJS Open*. 2022;6(4):zrac103. 10.1093/bjsopen/zrac103. 36029031 10.1093/bjsopen/zrac103 PMC9418812

[23] Howlader N, Ries LAG, Mariotto AB, Reichman ME, Ruhl J, Cronin KA. Improved estimates of cancer-specific survival rates from population-based data. *J Natl Cancer Inst*. 2010;102(20):1584–98. 10.1093/jnci/djq366. 20937991 10.1093/jnci/djq366 PMC2957430

[24] Cho H, Howlader N, Mariotto AB, Cronin KA. Estimating relative survival for cancer patients from the SEER Program using expected rates based on Ederer I versus Ederer II method. *Surveillance Research Program, National Cancer Institute*. Published online January 2011:1-17. https://surveillance.cancer.gov/reports/tech2011.01.pdf

[25] Carr NJ, Cecil TD, Mohamed F, et al. A consensus for classification and pathologic reporting of pseudomyxoma peritonei and associated appendiceal neoplasia: the results of the Peritoneal Surface Oncology Group International (PSOGI) Modified Delphi Process. *Am J Surg Pathol*. 2016;40(1):14–26. 10.1097/PAS.0000000000000535. 26492181 10.1097/PAS.0000000000000535

[26] Amin MB, Edge SB, Greene FL, et al. AJCC cancer staging manual. Springer International; 2018.

[27] Overman MJ, Fournier K, Hu CY, et al. Improving the AJCC/TNM staging for adenocarcinomas of the appendix: the prognostic impact of histological grade. *Ann Surg*. 2013. 10.1097/SLA.0b013e318269d680. 10.1097/SLA.0b013e318269d680 PMC3855996 23001080

[28] Davison JM, Choudry HA, Pingpank JF, et al. Clinicopathologic and molecular analysis of disseminated appendiceal mucinous neoplasms: Identification of factors predicting survival and proposed criteria for a three-tiered assessment of tumor grade. *Mod Pathol*. 2014;27(11):1521–39. 10.1038/modpathol.2014.37. 24633196 10.1038/modpathol.2014.37

[29] Chua TC, Al-Alem I, Saxena A, Liauw W, Morris DL. Surgical cytoreduction and survival in appendiceal cancer peritoneal carcinomatosis: an evaluation of 46 consecutive patients. *Ann Surg Oncol*. 2011;18(6):1540–6. 10.1245/s10434-011-1714-3. 21491165 10.1245/s10434-011-1714-3

[30] Baratti D, Kusamura S, Nonaka D, et al. Pseudomyxoma peritonei: clinical pathological and biological prognostic factors in patients treated with cytoreductive surgery and hyperthermic intraperitoneal chemotherapy (HIPEC). *Ann Surg Oncol*. 2008;15(2):526–34. 10.1245/s10434-007-9691-2. 18043976 10.1245/s10434-007-9691-2

[31] Sugarbaker PH, Bijelic L, Chang D, Yoo D. Neoadjuvant FOLFOX chemotherapy in 34 consecutive patients with mucinous peritoneal carcinomatosis of appendiceal origin. *J Surg Oncol*. 2010;102(6):576–81. 10.1002/jso.21679. 20737420 10.1002/jso.21679

[32] Turner KM, Hanna NN, Zhu Y, et al. Assessment of neoadjuvant chemotherapy on operative parameters and outcome in patients with peritoneal dissemination from high-grade appendiceal cancer. *Ann Surg Oncol*. 2013;20(4):1068–73. 10.1245/s10434-012-2789-1. 23456383 10.1245/s10434-012-2789-1

[33] Van Sweringen HL, Hanseman DJ, Ahmad SA, Edwards MJ, Sussman JJ. Predictors of survival in patients with high-grade peritoneal metastases undergoing cytoreductive surgery and hyperthermic intraperitoneal chemotherapy. *Surgery*. 2012;152(4):617–25. 10.1016/j.surg.2012.07.027. 22943843 10.1016/j.surg.2012.07.027

[34] Schomas DA, Miller RC, Donohue JH, et al. Intraperitoneal treatment for peritoneal mucinous carcinomatosis of appendiceal origin after operative management: Long-term follow-up of the Mayo Clinic experience. *Ann Surg*. 2009;249(4):588–95. 10.1097/SLA.0b013e31819ec7e3. 19300231 10.1097/SLA.0b013e31819ec7e3

[35] Turaga KK, Pappas SG, Gamblin TC. Importance of Histologic Subtype in the Staging of Appendiceal Tumors. *Ann surg oncol*. 2012;19(5):1379–85. 10.1245/s10434-012-2238-1. 22302267 10.1245/s10434-012-2238-1

[36] Ethnicity Work Group. *NAACCR Guideline for Enhancing Hispanic/Latino Identification: Revised NAACCR Hispanic/Latino Identification Algorithm [NHIA V2.2.1]*. North American Association of Central Cancer Registries; 2011:1-15. Accessed September 13, 2023. Available at: https://www.naaccr.org/wp-content/uploads/2016/11/NHIA_v2_2_1_09122011.pdf

[37] Levine EA, Stewart JHI, Russell GB, Geisinger KR, Loggie BL, Shen P. Cytoreductive surgery and intraperitoneal hyperthermic chemotherapy for peritoneal surface malignancy: experience with 501 procedures. *J Am Coll Surg*. 2007;204(5):943. 10.1016/j.jamcollsurg.2006.12.048. 17481516 10.1016/j.jamcollsurg.2006.12.048

[38] Smeenk RM, Verwaal VJ, Antonini N, Zoetmulder FAN. Survival analysis of pseudomyxoma peritonei patients treated by cytoreductive surgery and hyperthermic intraperitoneal chemotherapy. *Ann Surg*. 2007;245(1):104–9. 10.1097/01.sla.0000231705.40081.1a. 17197972 10.1097/01.sla.0000231705.40081.1a PMC1867935

[39] Khan F, Vogel RI, Diep GK, Tuttle TM, Lou E. Prognostic Factors for Survival in Advanced Appendiceal Cancers. *Cancer Biomark*. 2016;17(4):457–62. 10.3233/CBM-160662. 27802196 10.3233/CBM-160662 PMC5726252

[40] Grotz TE, Royal RE, Mansfield PF, et al. Stratification of outcomes for mucinous appendiceal adenocarcinoma with peritoneal metastasis by histological grade. *World J Gastrointest Oncol*. 2017;9(9):354–62. 10.4251/wjgo.v9.i9.354. 28979717 10.4251/wjgo.v9.i9.354 PMC5605335

[41] Santullo F, Pacelli F, Abatini C, et al. Cytoreduction and hyperthermic intraperitoneal chemotherapy for pseudomyxoma peritonei of appendiceal origin: a single center experience. *Front Surg*. 2021. 10.3389/fsurg.2021.715119. 10.3389/fsurg.2021.715119 PMC8427490 34513915

[42] Esquivel J, Piso P, Verwaal V, et al. American Society of peritoneal surface malignancies opinion statement on defining expectations from cytoreductive surgery and hyperthermic intraperitoneal chemotherapy in patients with colorectal cancer. *J Surg Oncol*. 2014;110(7):777–8. 10.1002/jso.23722. 25043759 10.1002/jso.23722

[43] Kusamura S, Bhatt A, Hubner M, et al. The 2022 PSOGI International Consensus on HIPEC Regimens for Peritoneal Malignancies: Methodology. *Ann Surg Oncol*. 2023;30(4):2508–19. 10.1245/s10434-022-12990-3. 36595113 10.1245/s10434-022-12990-3

[44] Adamo M, Groves, Dickie L, Ruhl J. SEER Program Coding and Staging Manual 2023. *National Cancer Institute, Bethesda, MD*. Published online September 2022:1-281.

[45] National Cancer Institute. Overview: Surveillance, Epidemiology, and End Results (SEER) Surveillance Research Program. Published online July 2021. Accessed March 17, 2024. https://seer.cancer.gov/about/factsheets/SEER_Overview.pdf

