# Supplementary material for: Predicting Survival in Mucinous Adenocarcinoma of the Appendix: Demographics, Disease Presentation, and Treatment Methodology
Source: Ann Surg Oncol. 2024 Jun 14;31(9):6237–51. doi: 10.1245/s10434-024-15526-z (PMC11300641; doi:10.1245/s10434-024-15526-z)
Supplement: Supplementary file 1 — Supplementary file1 Supplementary Table 1 Incidence of demographic attributes and presenting characteristics in patients with mucinous adenocarcinoma of the appendix (MACA) (17 KB) [file 10434_2024_15526_MOESM1_ESM.docx]

**Supplementary Table 1 –** Incidence of demographic attributes and presenting characteristics in patients with mucinous adenocarcinoma of the appendix (MACA)

| **Variable** | Total  (*n* = 4,258) | 2005  (*n* = 191) | 2006  (*n* = 224) | 2007  (*n* = 202) | 2008  (*n* = 257) | 2009  (*n* = 233) | 2010  (*n* = 290) | 2011  (*n* = 275) | 2012  (*n* = 286) | 2013  (*n* = 308) | 2014  (*n* = 325) | 2015  (*n* = 332) | 2016  (*n* = 314) | 2017  (*n* = 371) | 2018  (*n* = 306) | 2019  (*n* = 344) | ***p value*** |
| --- | --- | --- | --- | --- | --- | --- | --- | --- | --- | --- | --- | --- | --- | --- | --- | --- | --- |
| **Age** |  |  |  |  |  |  |  |  |  |  |  |  |  |  |  |  | 0.052 |
| 10-19 years | 3 (0.1%) | 1 (0.5%) | 1 (0.4%) | 0 (0.0%) | 0 (0.0%) | 0 (0.0%) | 0 (0.0%) | 0 (0.0%) | 0 (0.0%) | 1 (0.3%) | 0 (0.0%) | 0 (0.0%) | 0 (0.0%) | 0 (0.0%) | 0 (0.0%) | 0 (0.0%) |  |
| 20-29 years | 61 (1.4%) | 2 (1.0%) | 2 (0.9%) | 1 (0.5%) | 1 (0.4%) | 5 (2.1%) | 2 (0.7%) | 5 (1.8%) | 8 (2.8%) | 2 (0.6%) | 5 (1.5%) | 6 (1.8%) | 8 (2.5%) | 3 (0.8%) | 5 (1.6%) | 6 (1.7%) |  |
| 30-39 years | 268 (6.3%) | 12 (6.3%) | 17 (7.6%) | 8 (4.0%) | 13 (5.1%) | 16 (6.9%) | 18 (6.2%) | 13 (4.7%) | 19 (6.6%) | 19 (6.2%) | 26 (8.0%) | 19 (5.7%) | 23 (7.3%) | 24 (6.5%) | 16 (5.2%) | 25 (7.3%) |  |
| 40-49 years | 660 (15.5%) | 43 (22.5%) | 36 (16.1%) | 38 (18.8%) | 45 (17.5%) | 41 (17.6%) | 49 (16.9%) | 53 (19.3%) | 47 (16.4%) | 51 (16.6%) | 40 (12.3%) | 50 (15.1%) | 43 (13.7%) | 52 (14.0%) | 37 (12.1%) | 35 (10.2%) |  |
| 50-59 years | 1,133 (26.6%) | 53 (27.7%) | 68 (30.4%) | 61 (30.2%) | 66 (25.7%) | 71 (30.5%) | 94 (32.4%) | 75 (27.3%) | 72 (25.2%) | 69 (22.4%) | 98 (30.2%) | 90 (27.1%) | 74 (23.6%) | 71 (19.1%) | 80 (26.1%) | 91 (26.5%) |  |
| 60-69 years | 1,083 (25.4%) | 37 (19.4%) | 50 (22.3%) | 48 (23.8%) | 67 (26.1%) | 47 (20.2%) | 60 (20.7%) | 69 (25.1%) | 68 (23.8%) | 78 (25.3%) | 84 (25.8%) | 96 (28.9%) | 93 (29.6%) | 113 (30.5%) | 81 (26.5%) | 92 (26.7%) |  |
| 70-79 years | 732 (17.2%) | 27 (14.1%) | 33 (14.7%) | 31 (15.3%) | 44 (17.1%) | 35 (15.0%) | 47 (16.2%) | 45 (16.4%) | 52 (18.2%) | 66 (21.4%) | 51 (15.7%) | 48 (14.5%) | 49 (15.6%) | 76 (20.5%) | 55 (18.0%) | 73 (21.2%) |  |
| 80+ years | 318 (7.5%) | 16 (8.4%) | 17 (7.6%) | 15 (7.4%) | 21 (8.2%) | 18 (7.7%) | 20 (6.9%) | 15 (5.5%) | 20 (7.0%) | 22 (7.1%) | 21 (6.5%) | 23 (6.9%) | 24 (7.6%) | 32 (8.6%) | 32 (10.5%) | 22 (6.4%) |  |
| **Sex** |  |  |  |  |  |  |  |  |  |  |  |  |  |  |  |  | 0.422 |
| Female | 2,380 (55.9%) | 101 (52.9%) | 127 (56.7%) | 106 (52.5%) | 140 (54.5%) | 143 (61.4%) | 160 (55.2%) | 161 (58.5%) | 141 (49.3%) | 168 (54.5%) | 184 (56.6%) | 194 (58.4%) | 167 (53.2%) | 211 (56.9%) | 181 (59.2%) | 196 (57.0%) |  |
| Male | 1,878 (44.1%) | 90 (47.1%) | 97 (43.3%) | 96 (47.5%) | 117 (45.5%) | 90 (38.6%) | 130 (44.8%) | 114 (41.5%) | 145 (50.7%) | 140 (45.5%) | 141 (43.4%) | 138 (41.6%) | 147 (46.8%) | 160 (43.1%) | 125 (40.8%) | 148 (43.0%) |  |
| **Race/Ethnicity** |  |  |  |  |  |  |  |  |  |  |  |  |  |  |  |  | 0.058 |
| Non-Hispanic White | 2,901 (68.1%) | 132 (69.1%) | 153 (68.3%) | 140 (69.3%) | 197 (76.7%) | 168 (72.1%) | 204 (70.3%) | 205 (74.5%) | 189 (66.1%) | 215 (69.8%) | 217 (66.8%) | 221 (66.6%) | 205 (65.3%) | 231 (62.3%) | 206 (67.3%) | 218 (63.4%) |  |
| Hispanic (All Races) | 596 (14.0%) | 25 (13.1%) | 36 (16.1%) | 27 (13.4%) | 25 (9.7%) | 22 (9.4%) | 34 (11.7%) | 31 (11.3%) | 52 (18.2%) | 47 (15.3%) | 41 (12.6%) | 45 (13.6%) | 47 (15.0%) | 63 (17.0%) | 48 (15.7%) | 53 (15.4%) |  |
| Non-Hispanic Black | 370 (8.7%) | 14 (7.3%) | 17 (7.6%) | 17 (8.4%) | 17 (6.6%) | 21 (9.0%) | 29 (10.0%) | 22 (8.0%) | 23 (8.0%) | 16 (5.2%) | 30 (9.2%) | 36 (10.8%) | 26 (8.3%) | 44 (11.9%) | 26 (8.5%) | 32 (9.3%) |  |
| Non-Hispanic Asian or Pacific Islander | 355 (8.3%) | 18 (9.4%) | 17 (7.6%) | 18 (8.9%) | 18 (7.0%) | 21 (9.0%) | 23 (7.9%) | 14 (5.1%) | 19 (6.6%) | 28 (9.1%) | 33 (10.2%) | 29 (8.7%) | 32 (10.2%) | 29 (7.8%) | 24 (7.8%) | 32 (9.3%) |  |
| Non-Hispanic Other Race | 36 (0.8%) | 2 (1.0%) | 1 (0.4%) | 0 (0.0%) | 0 (0.0%) | 1 (0.4%) | 0 (0.0%) | 3 (1.1%) | 3 (1.0%) | 2 (0.6%) | 4 (1.2%) | 1 (0.3%) | 4 (1.3%) | 4 (1.1%) | 2 (0.7%) | 9 (2.6%) |  |
| **U.S. Census Region** |  |  |  |  |  |  |  |  |  |  |  |  |  |  |  |  | 0.395 |
| Northeast | 819 (19.2%) | 38 (19.9%) | 34 (15.2%) | 35 (17.3%) | 54 (21.0%) | 42 (18.0%) | 72 (24.8%) | 56 (20.4%) | 54 (18.9%) | 62 (20.1%) | 58 (17.8%) | 59 (17.8%) | 60 (19.1%) | 75 (20.2%) | 59 (19.3%) | 61 (17.7%) |  |
| South | 956 (22.5%) | 38 (19.9%) | 42 (18.8%) | 46 (22.8%) | 62 (24.1%) | 53 (22.7%) | 57 (19.7%) | 62 (22.5%) | 64 (22.4%) | 68 (22.1%) | 65 (20.0%) | 78 (23.5%) | 61 (19.4%) | 90 (24.3%) | 73 (23.9%) | 97 (28.2%) |  |
| Midwest | 150 (3.5%) | 10 (5.2%) | 5 (2.2%) | 4 (2.0%) | 10 (3.9%) | 9 (3.9%) | 10 (3.4%) | 8 (2.9%) | 17 (5.9%) | 14 (4.5%) | 15 (4.6%) | 9 (2.7%) | 14 (4.5%) | 8 (2.2%) | 9 (2.9%) | 8 (2.3%) |  |
| West | 2,333 (54.8%) | 105 (55.0%) | 143 (63.8%) | 117 (57.9%) | 131 (51.0%) | 129 (55.4%) | 151 (52.1%) | 149 (54.2%) | 151 (52.8%) | 164 (53.2%) | 187 (57.5%) | 186 (56.0%) | 179 (57.0%) | 198 (53.4%) | 165 (53.9%) | 178 (51.7%) |  |
| **Urban/Rural Classification** |  |  |  |  |  |  |  |  |  |  |  |  |  |  |  |  | 0.210 |
| Urban (pop. > 1,000,000) | 2,617 (61.5%) | 114 (59.7%) | 137 (61.2%) | 132 (65.3%) | 160 (62.3%) | 145 (62.2%) | 176 (60.7%) | 169 (61.5%) | 174 (60.8%) | 162 (52.6%) | 221 (68.0%) | 217 (65.4%) | 203 (64.6%) | 214 (57.8%) | 173 (56.5%) | 220 (64.3%) |  |
| Urban (pop. 250,000-1,000,000) | 882 (20.7%) | 43 (22.5%) | 47 (21.0%) | 33 (16.3%) | 53 (20.6%) | 44 (18.9%) | 67 (23.1%) | 62 (22.5%) | 60 (21.0%) | 70 (22.7%) | 55 (16.9%) | 61 (18.4%) | 63 (20.1%) | 95 (25.7%) | 76 (24.8%) | 53 (15.5%) |  |
| Urban (pop. < 250,000) | 319 (7.5%) | 18 (9.4%) | 16 (7.1%) | 19 (9.4%) | 19 (7.4%) | 15 (6.4%) | 19 (6.6%) | 21 (7.6%) | 22 (7.7%) | 34 (11.0%) | 12 (3.7%) | 22 (6.6%) | 20 (6.4%) | 26 (7.0%) | 23 (7.5%) | 33 (9.6%) |  |
| Rural (urban-adjacent) | 252 (5.9%) | 10 (5.2%) | 15 (6.7%) | 12 (5.9%) | 15 (5.8%) | 18 (7.7%) | 13 (4.5%) | 11 (4.0%) | 20 (7.0%) | 27 (8.8%) | 24 (7.4%) | 15 (4.5%) | 15 (4.8%) | 21 (5.7%) | 17 (5.6%) | 19 (5.6%) |  |
| Rural | 185 (4.3%) | 6 (3.1%) | 9 (4.0%) | 6 (3.0%) | 10 (3.9%) | 11 (4.7%) | 15 (5.2%) | 12 (4.4%) | 10 (3.5%) | 15 (4.9%) | 13 (4.0%) | 17 (5.1%) | 13 (4.1%) | 14 (3.8%) | 17 (5.6%) | 17 (5.0%) |  |
| **Median Household Income** |  |  |  |  |  |  |  |  |  |  |  |  |  |  |  |  | **< 0.001** |
| More than $75,000 | 1,431 (33.6%) | 80 (41.9%) | 73 (32.6%) | 61 (30.2%) | 74 (28.8%) | 61 (26.2%) | 101 (34.8%) | 80 (29.1%) | 88 (30.8%) | 88 (28.6%) | 108 (33.2%) | 112 (33.7%) | 128 (40.8%) | 132 (35.6%) | 114 (37.3%) | 131 (38.1%) |  |
| $65,000 - $74,999 | 1,097 (25.8%) | 45 (23.6%) | 85 (37.9%) | 81 (40.1%) | 100 (38.9%) | 55 (23.6%) | 56 (19.3%) | 60 (21.8%) | 56 (19.6%) | 63 (20.5%) | 54 (16.6%) | 55 (16.6%) | 93 (29.6%) | 103 (27.8%) | 86 (28.1%) | 105 (30.5%) |  |
| $55,000 - $64,999 | 875 (20.5%) | 43 (22.5%) | 22 (9.8%) | 24 (11.9%) | 35 (13.6%) | 68 (29.2%) | 81 (27.9%) | 72 (26.2%) | 75 (26.2%) | 80 (26.0%) | 92 (28.3%) | 93 (28.0%) | 46 (14.6%) | 56 (15.1%) | 44 (14.4%) | 44 (12.8%) |  |
| $45,000 - $54,999 | 537 (12.6%) | 18 (9.4%) | 30 (13.4%) | 23 (11.4%) | 26 (10.1%) | 32 (13.7%) | 31 (10.7%) | 37 (13.5%) | 38 (13.3%) | 48 (15.6%) | 44 (13.5%) | 45 (13.6%) | 28 (8.9%) | 60 (16.2%) | 38 (12.4%) | 39 (11.3%) |  |
| $35,000 - $44,999 | 254 (6.0%) | 3 (1.6%) | 12 (5.4%) | 11 (5.4%) | 16 (6.2%) | 15 (6.4%) | 16 (5.5%) | 23 (8.4%) | 27 (9.4%) | 23 (7.5%) | 19 (5.8%) | 23 (6.9%) | 14 (4.5%) | 14 (3.8%) | 19 (6.2%) | 19 (5.5%) |  |
| Less than $35,000 | 64 (1.5%) | 2 (1.0%) | 2 (0.9%) | 2 (1.0%) | 6 (2.3%) | 2 (0.9%) | 5 (1.7%) | 3 (1.1%) | 2 (0.7%) | 6 (1.9%) | 8 (2.5%) | 4 (1.2%) | 5 (1.6%) | 6 (1.6%) | 5 (1.6%) | 6 (1.7%) |  |
| **Histopathologic Grade** |  |  |  |  |  |  |  |  |  |  |  |  |  |  |  |  | **0.019** |
| Well-differentiated - Grade 1 | 1,260 (36.6%) | 49 (32.7%) | 57 (33.1%) | 55 (36.9%) | 79 (38.9%) | 57 (32.6%) | 92 (36.8%) | 83 (34.4%) | 72 (30.1%) | 102 (38.6%) | 89 (32.2%) | 90 (33.0%) | 98 (36.3%) | 124 (41.8%) | 92 (41.4%) | 121 (45.7%) |  |
| Moderately differentiated - Grade 2 | 1,142 (33.1%) | 53 (35.3%) | 48 (27.9%) | 42 (28.2%) | 57 (28.1%) | 55 (31.4%) | 84 (33.6%) | 81 (33.6%) | 91 (38.1%) | 87 (33.0%) | 97 (35.1%) | 98 (35.9%) | 96 (35.6%) | 103 (34.7%) | 66 (29.7%) | 84 (31.7%) |  |
| Poorly differentiated - Grade 3 | 1,044 (30.3%) | 48 (32.0%) | 67 (39.0%) | 52 (34.9%) | 67 (33.0%) | 63 (36.0%) | 74 (29.6%) | 77 (32.0%) | 76 (31.8%) | 75 (28.4%) | 90 (32.6%) | 85 (31.1%) | 76 (28.1%) | 70 (23.6%) | 64 (28.8%) | 60 (22.6%) |  |
| **Lymph Node Status** |  |  |  |  |  |  |  |  |  |  |  |  |  |  |  |  | 0.083 |
| Negative | 1,863 (71.1%) | 77 (67.0%) | 97 (63.8%) | 84 (65.6%) | 105 (68.6%) | 96 (72.7%) | 115 (67.6%) | 130 (70.7%) | 131 (76.2%) | 139 (69.5%) | 130 (66.7%) | 148 (72.2%) | 161 (75.9%) | 172 (74.1%) | 132 (71.7%) | 146 (78.9%) |  |
| Positive | 756 (28.9%) | 38 (33.0%) | 55 (36.2%) | 44 (34.4%) | 48 (31.4%) | 36 (27.3%) | 55 (32.4%) | 54 (29.3%) | 41 (23.8%) | 61 (30.5%) | 65 (33.3%) | 57 (27.8%) | 51 (24.1%) | 60 (25.9%) | 52 (28.3%) | 39 (21.1%) |  |
| **Combined Summary Stage** |  |  |  |  |  |  |  |  |  |  |  |  |  |  |  |  | **< 0.001** |
| Localized | 683 (16.5%) | 42 (22.6%) | 44 (20.5%) | 37 (19.1%) | 60 (24.0%) | 44 (19.5%) | 39 (13.8%) | 45 (16.8%) | 45 (16.3%) | 53 (17.5%) | 48 (15.3%) | 38 (11.8%) | 48 (15.4%) | 52 (14.2%) | 39 (13.1%) | 49 (14.6%) |  |
| Regional | 980 (23.6%) | 51 (27.4%) | 64 (29.8%) | 51 (26.3%) | 54 (21.6%) | 54 (23.9%) | 55 (19.5%) | 66 (24.6%) | 57 (20.7%) | 63 (20.8%) | 70 (22.4%) | 63 (19.6%) | 93 (29.8%) | 86 (23.5%) | 74 (24.9%) | 79 (23.6%) |  |
| Distant | 2,481 (59.9%) | 93 (50.0%) | 107 (49.8%) | 106 (54.6%) | 136 (54.4%) | 128 (56.6%) | 188 (66.7%) | 157 (58.6%) | 174 (63.0%) | 187 (61.7%) | 195 (62.3%) | 220 (68.5%) | 171 (54.8%) | 228 (62.3%) | 184 (62.0%) | 207 (61.8%) |  |
