# Supplementary material for: Predicting Survival in Mucinous Adenocarcinoma of the Appendix: Demographics, Disease Presentation, and Treatment Methodology
Source: Ann Surg Oncol. 2024 Jun 14;31(9):6237–51. doi: 10.1245/s10434-024-15526-z (PMC11300641; doi:10.1245/s10434-024-15526-z)
Supplement: Supplementary file 2 — Supplementary file2 Supplementary Table 2 Comparison of median disease-specific survival and overall survival for patients with mucinous adenocarcinoma of the appendix (MACA) (10 KB) [file 10434_2024_15526_MOESM2_ESM.docx]

**Supplementary Table 2 –** Comparison of median disease-specific survival and overall survival for patients with mucinous adenocarcinoma of the appendix (MACA)

| **Variable** | ***n (%)*** | **Median DSS (95% CI), months** | **log-rank *p*** | **Median OS (95% CI), months** | **log-rank *p*** |
| --- | --- | --- | --- | --- | --- |
| **All** | 3,222 (100.0%) | 118 (103, 138) |  | 88 (79, 102) |  |
| **Age, by decade** |  |  | **< 0.001** |  | **< 0.001** |
| 20-29 years | 55 (1.7%) | 78 (61, NA) |  | 78 (44, NA) |  |
| 30-39 years | 240 (7.4%) | NA (NA, NA) |  | NA (NA, NA) |  |
| 40-49 years | 598 (18.6%) | 118 (95, NA) |  | 107 (79, 149) |  |
| 50-59 years | 961 (29.8%) | 102 (85, 146) |  | 91 (76, 114) |  |
| 60-69 years | 847 (26.3%) | 116 (80, NA) |  | 88 (71, 116) |  |
| 70-79 years | 521 (16.2%) | 103 (61, NA) |  | 53 (50, 62) |  |
| **Sex** |  |  | 0.307 |  | **0.036** |
| Female | 1,818 (56.4%) | 144 (117, NA) |  | 107 (87, 132) |  |
| Male | 1,404 (43.6%) | 98 (80, 126) |  | 76 (67, 89) |  |
| **Race/ethnicity** |  |  | 0.110 |  | 0.122 |
| Non-Hispanic White | 2,119 (65.8%) | 125 (98, 153) |  | 88 (78, 108) |  |
| Hispanic (All Races) | 488 (15.1%) | 133 (109, NA) |  | 112 (83, 151) |  |
| Non-Hispanic Black | 298 (9.2%) | 129 (79, NA) |  | 82 (43, 127) |  |
| Non-Hispanic Asian or Pacific Islander | 284 (8.8%) | 75 (56, 125) |  | 65 (54, 102) |  |
| Non-Hispanic Other Race | 33 (1.0%) | NA (NA, NA) |  | NA (NA, NA) |  |
| **Year of diagnosis** |  |  | 0.733 |  | 0.967 |
| 2005-2009 | 822 (25.5%) | 118 (92, 146) |  | 89 (72, 114) |  |
| 2010-2014 | 1,099 (34.1%) | 110 (88, NA) |  | 87 (79, 106) |  |
| 2015-2019 | 1,301 (40.4%) | NA (NA, NA) |  | NA (NA, NA) |  |
| **U.S. Census region** |  |  | **0.008** |  | **0.004** |
| Northeast | 609 (18.9%) | NA (NA, NA) |  | 138 (95, NA) |  |
| South | 728 (22.6%) | 106 (82, NA) |  | 79 (57, 100) |  |
| Midwest | 105 (3.3%) | 63 (50, NA) |  | 59 (49, 94) |  |
| West | 1,780 (55.2%) | 110 (92, 129) |  | 85 (76, 106) |  |
| **Urban/rural classification** |  |  | **0.018** |  | **0.003** |
| Urban (pop. > 1,000,000) | 1,992 (61.9%) | 129 (110, NA) |  | 102 (82, 125) |  |
| Urban (pop. 250,000-1,000,000) | 666 (20.7%) | 127 (89, NA) |  | 91 (76, 127) |  |
| Urban (pop. < 250,000) | 240 (7.5%) | 95 (56, 153) |  | 61 (52, 87) |  |
| Rural (urban-adjacent) | 184 (5.7%) | 67 (48, NA) |  | 53 (46, 123) |  |
| Rural | 137 (4.3%) | 87 (52, NA) |  | 77 (41, 112) |  |
| **Median household income** |  |  | **0.043** |  | **0.007** |
| More than $75,000 | 1,091 (33.9%) | 126 (93, 149) |  | 92 (78, 126) |  |
| $65,000 - $74,999 | 833 (25.9%) | 120 (95, NA) |  | 101 (76, 129) |  |
| $55,000 - $64,999 | 661 (20.5%) | 133 (98, NA) |  | 97 (75, 133) |  |
| $45,000 - $54,999 | 395 (12.3%) | 95 (65, NA) |  | 76 (56, 95) |  |
| $35,000 - $44,999 | 189 (5.9%) | 79 (41, NA) |  | 51 (33, 87) |  |
| Less than $35,000 | 53 (1.6%) | 114 (46, NA) |  | 114 (42, NA) |  |
| **Histopathologic grade** |  |  | **< 0.001** |  | **< 0.001** |
| Well-differentiated - Grade 1 | 962 (37.0%) | NA (NA, NA) |  | NA (NA, NA) |  |
| Moderately differentiated - Grade 2 | 864 (33.2%) | NA (133, NA) |  | 129 (99, NA) |  |
| Poorly differentiated - Grade 3 | 777 (29.9%) | 32 (28, 35) |  | 29 (26, 33) |  |
| **Lymph node status** |  |  | **< 0.001** |  | **< 0.001** |
| Negative | 1,414 (70.8%) | NA (NA, NA) |  | NA (NA, NA) |  |
| Positive | 583 (29.2%) | 31 (27, 34) |  | 29 (26, 33) |  |
| **Combined summary stage** |  |  | **< 0.001** |  | **< 0.001** |
| Localized | 475 (15.1%) | NA (NA, NA) |  | NA (NA, NA) |  |
| Regional | 688 (21.9%) | NA (NA, NA) |  | NA (NA, NA) |  |
| Distant | 1,976 (62.9%) | 53 (50, 64) |  | 49 (44, 53) |  |

DSS = disease-specific survival

OS = overall survival
