# Supplementary material for: Predicting Survival in Mucinous Adenocarcinoma of the Appendix: Demographics, Disease Presentation, and Treatment Methodology
Source: Ann Surg Oncol. 2024 Jun 14;31(9):6237–51. doi: 10.1245/s10434-024-15526-z (PMC11300641; doi:10.1245/s10434-024-15526-z)
Supplement: Supplementary file 3 — Supplementary file3 Supplementary Table 3 Comparison of median disease-specific survival and overall survival for patients with mucinous adenocarcinoma of the appendix (MACA) and cytoreductive surgery with hyperthermic intraperitoneal chemotherapy (CRS-HIPEC) rationale (11 KB) [file 10434_2024_15526_MOESM3_ESM.docx]

**Supplementary Table 3 –** Comparison of median disease-specific survival and overall survival for patients with mucinous adenocarcinoma of the appendix (MACA) and cytoreductive surgery with hyperthermic intraperitoneal chemotherapy (CRS-HIPEC) rationale

| **Variable** | ***n (%)*** | **Median DSS (95% CI), months** | **log-rank *p*** | **Median OS (95% CI), months** | **log-rank *p*** |
| --- | --- | --- | --- | --- | --- |
| **All** | 2,387 (100.0%) |  |  |  |  |
| **Age, by decade** |  |  | **0.001** |  | **< 0.001** |
| 20-29 years | 40 (1.7%) | 72 (36, NA) |  | 72 (36, NA) |  |
| 30-39 years | 181 (7.6%) | NA (NA, NA) |  | NA (NA, NA) |  |
| 40-49 years | 461 (19.3%) | 98 (74, 138) |  | 80 (63, 118) |  |
| 50-59 years | 727 (30.5%) | 87 (72, 120) |  | 82 (65, 101) |  |
| 60-69 years | 608 (25.5%) | 87 (75, 151) |  | 79 (61, 101) |  |
| 70-79 years | 370 (15.5%) | 69 (53, NA) |  | 53 (44, 61) |  |
| **Sex** |  |  | 0.999 |  | 0.303 |
| Female | 1,421 (59.5%) | 107 (82, 138) |  | 84 (65, 110) |  |
| Male | 966 (40.5%) | 87 (76, 108) |  | 75 (64, 83) |  |
| **Race/ethnicity** |  |  | 0.129 |  | 0.119 |
| Non-Hispanic White | 1,568 (65.7%) | 95 (79, 119) |  | 78 (65, 90) |  |
| Hispanic (All Races) | 367 (15.4%) | 120 (99, NA) |  | 109 (75, 135) |  |
| Non-Hispanic Black | 210 (8.8%) | 82 (40, NA) |  | 51 (37, 123) |  |
| Non-Hispanic Asian or Pacific Islander | 220 (9.2%) | 65 (53, 104) |  | 62 (49, 100) |  |
| Non-Hispanic Other Race | 22 (0.9%) | 78 (46, NA) |  | 78 (46, NA) |  |
| **Year of diagnosis** |  |  | **0.040** |  | 0.148 |
| 2005-2009 | 589 (24.7%) | 75 (59, 101) |  | 65 (53, 78) |  |
| 2010-2014 | 832 (34.9%) | 100 (80, NA) |  | 81 (69, 100) |  |
| 2015-2019 | 966 (40.5%) | NA (NA, NA) |  | NA (NA, NA) |  |
| **U.S. Census region** |  |  | **0.006** |  | **0.005** |
| Northeast | 444 (18.6%) | 142 (126, NA) |  | 126 (78, NA) |  |
| South | 529 (22.2%) | 79 (52, 100) |  | 55 (47, 82) |  |
| Midwest | 79 (3.3%) | 60 (50, NA) |  | 58 (46, 91) |  |
| West | 1,335 (55.9%) | 98 (79, 117) |  | 80 (71, 100) |  |
| **Urban/rural classification** |  |  | **0.005** |  | **0.003** |
| Urban (pop. > 1,000,000) | 1,477 (61.9%) | 109 (85, 138) |  | 82 (74, 106) |  |
| Urban (pop. 250,000-1,000,000) | 499 (20.9%) | 112 (76, 151) |  | 87 (64, 126) |  |
| Urban (pop. < 250,000) | 174 (7.3%) | 62 (52, 106) |  | 57 (45, 80) |  |
| Rural (urban-adjacent) | 138 (5.8%) | 50 (41, NA) |  | 49 (36, 108) |  |
| Rural | 97 (4.1%) | 55 (36, NA) |  | 55 (35, 97) |  |
| **Median household income** |  |  | **0.009** |  | **0.006** |
| More than $75,000 | 816 (34.2%) | 104 (79, 146) |  | 83 (63, 116) |  |
| $65,000 - $74,999 | 608 (25.5%) | 102 (76, 138) |  | 80 (66, 109) |  |
| $55,000 - $64,999 | 495 (20.7%) | 100 (75, NA) |  | 82 (69, 118) |  |
| $45,000 - $54,999 | 293 (12.3%) | 79 (53, 117) |  | 61 (52, 94) |  |
| $35,000 - $44,999 | 137 (5.7%) | 41 (26, 108) |  | 35 (26, 79) |  |
| Less than $35,000 | 38 (1.6%) | 114 (42, NA) |  | 114 (25, NA) |  |
| **Histopathologic grade** |  |  | **< 0.001** |  | **< 0.001** |
| Well-differentiated - Grade 1 | 726 (35.4%) | NA (NA, NA) |  | NA (NA, NA) |  |
| Moderately differentiated - Grade 2 | 690 (33.6%) | 133 (99, NA) |  | 116 (87, 138) |  |
| Poorly differentiated - Grade 3 | 635 (31.0%) | 31 (27, 33) |  | 28 (24, 32) |  |
| **Lymph node status** |  |  | **< 0.001** |  | **< 0.001** |
| Negative | 1,070 (64.9%) | NA (NA, NA) |  | 138 (123, NA) |  |
| Positive | 578 (35.1%) | 31 (28, 35) |  | 29 (26, 33) |  |
| **Combined summary stage** |  |  | **< 0.001** |  | **< 0.001** |
| Regional | 678 (28.4%) | NA (NA, NA) |  | NA (NA, NA) |  |
| Distant | 1,709 (71.6%) | 71 (57, 80) |  | 59 (52, 71) |  |
| **Time to treatment** |  |  | **0.013** |  | 0.087 |
| Less than 1 month | 1,783 (74.9%) | 116 (90, 142) |  | 84 (76, 101) |  |
| 1-2 months | 342 (14.4%) | 56 (47, 80) |  | 53 (46, 74) |  |
| 2-3 months | 137 (5.8%) | 85 (60, NA) |  | 76 (53, 106) |  |
| More than 3 months | 120 (5.0%) | 75 (49, NA) |  | 69 (44, NA) |  |
| **Treatment strategy** |  |  | **< 0.001** |  | **< 0.001** |
| Surgery only | 881 (36.9%) | 126 (98, NA) |  | 89 (76, 117) |  |
| Surgery and other systemic therapy | 1,152 (48.3%) | 57 (50, 76) |  | 53 (48, 65) |  |
| CRS-HIPEC only | 217 (9.1%) | NA (NA, NA) |  | 116 (106, NA) |  |
| CRS-HIPEC with systemic therapy | 137 (5.7%) | 104 (53, NA) |  | 104 (53, NA) |  |

DSS = disease-specific survival

OS = overall survival
