# Supplementary material for: Predicting Survival in Mucinous Adenocarcinoma of the Appendix: Demographics, Disease Presentation, and Treatment Methodology
Source: Ann Surg Oncol. 2024 Jun 14;31(9):6237–51. doi: 10.1245/s10434-024-15526-z (PMC11300641; doi:10.1245/s10434-024-15526-z)
Supplement: Supplementary file 4 — Supplementary file4 Supplementary Fig. 1 Flow diagram of cohort selection, with inclusion and exclusion criteria (62 KB) [file 10434_2024_15526_MOESM4_ESM.pdf]

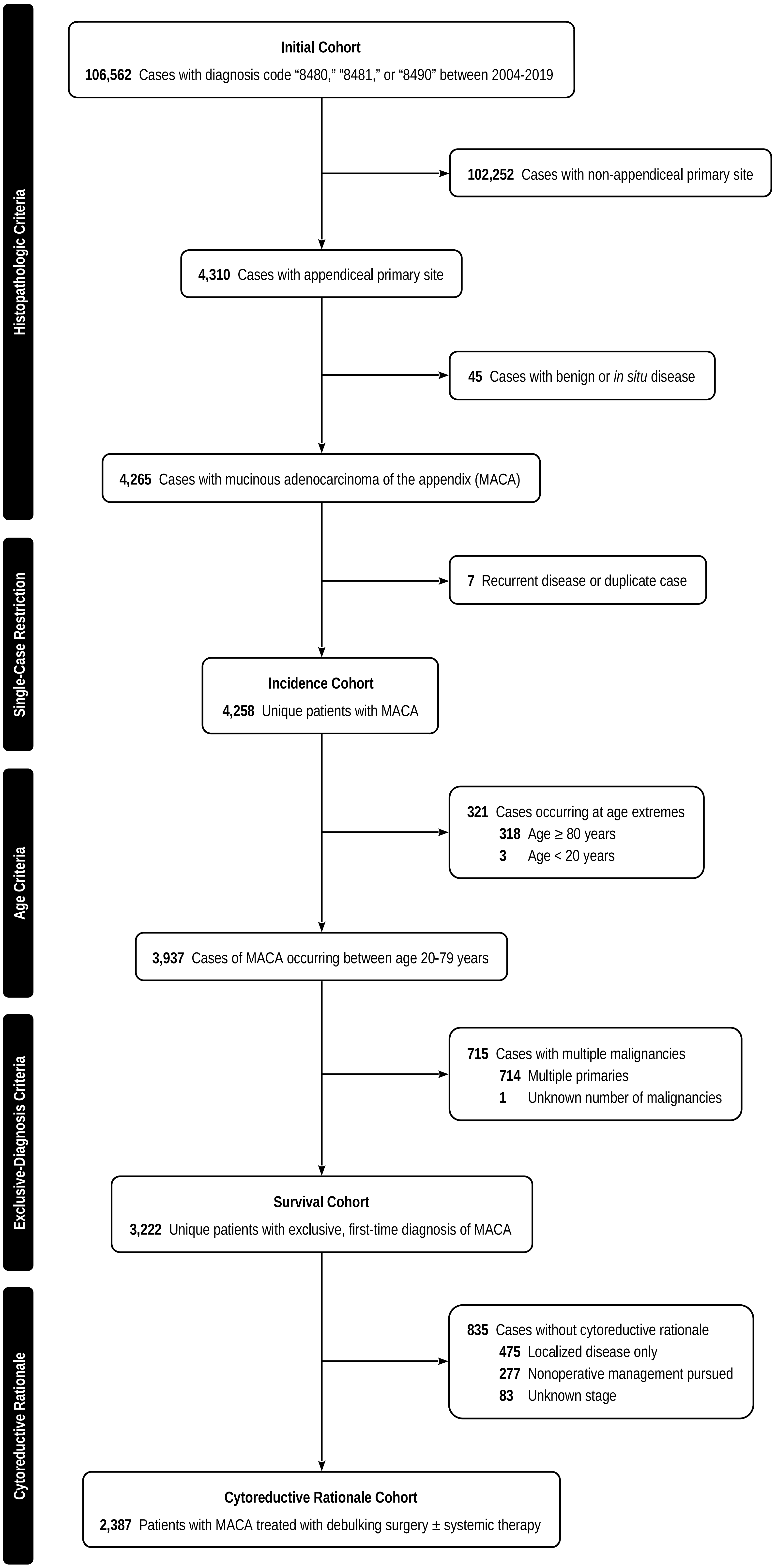

Supplementary Figure 1 – Flow diagram of cohort selection, with inclusion and exclusion criteria
