# Supplementary material for: Predicting Survival in Mucinous Adenocarcinoma of the Appendix: Demographics, Disease Presentation, and Treatment Methodology
Source: Ann Surg Oncol. 2024 Jun 14;31(9):6237–51. doi: 10.1245/s10434-024-15526-z (PMC11300641; doi:10.1245/s10434-024-15526-z)
Supplement: Supplementary file 5 — Supplementary file5 Supplementary Fig. 2 Multivariable Cox proportional hazards model for overall survival (OS), survival cohort (90 KB) [file 10434_2024_15526_MOESM5_ESM.pdf]

Variable	Cohort	HR (95% CI; *p*-value)
**Age, by decade**	20-29 years	-
	30-39 years	0.61 (0.32-1.16; $p = 0.133$)
	40-49 years	0.71 (0.39-1.26; $p = 0.241$)
	50-59 years	0.68 (0.39-1.22; $p = 0.197$)
	60-69 years	0.68 (0.38-1.22; $p = 0.199$)
	70-79 years	1.00 (0.55-1.79; $p = 0.991$)
**Sex**	Female	-
	Male	1.16 (0.99-1.36; $p = 0.062$)
**Race/ethnicity**	Non-Hispanic White	-
	Hispanic (All Races)	1.28 (1.00-1.64; $p = 0.048$)
	Non-Hispanic Black	1.37 (1.05-1.79; $p = 0.022$)
	Non-Hispanic Asian or Pacific Islander	1.20 (0.91-1.59; $p = 0.206$)
	Non-Hispanic Other Race	0.89 (0.28-2.82; $p = 0.845$)
**Year of diagnosis**	2005-2009	-
	2010-2014	0.79 (0.66-0.95; $p = 0.011$)
	2015-2019	0.82 (0.66-1.03; $p = 0.084$)
**U.S. Census region**	Northeast	-
	South	1.31 (0.98-1.75; $p = 0.071$)
	Midwest	1.47 (0.93-2.31; $p = 0.096$)
	West	1.16 (0.93-1.45; $p = 0.187$)
**Urban/rural classification**	Urban (pop. > 1,000,000)	-
	Urban (pop. 250,000-1,000,000)	1.07 (0.87-1.31; $p = 0.516$)
	Urban (pop. < 250,000)	1.34 (0.97-1.85; $p = 0.074$)
	Rural (urban-adjacent)	1.23 (0.84-1.81; $p = 0.287$)
	Rural	1.28 (0.74-2.20; $p = 0.381$)
**Median household income**	More than $75,000	-
	$65,000 - $74,999	1.06 (0.86-1.31; $p = 0.567$)
	$55,000 - $64,999	1.16 (0.92-1.45; $p = 0.207$)
	$45,000 - $54,999	1.09 (0.79-1.50; $p = 0.589$)
	$35,000 - $44,999	1.35 (0.86-2.11; $p = 0.192$)
	Less than $35,000	1.05 (0.47-2.34; $p = 0.897$)
**Histopathologic grade**	Well-differentiated - Grade 1	-
	Moderately differentiated - Grade 2	1.50 (1.20-1.87; $p < 0.001$)
	Poorly differentiated - Grade 3	2.88 (2.32-3.58; $p < 0.001$)
**Lymph node status**	Negative	-
	Positive	2.48 (2.07-2.96; $p < 0.001$)
**Combined summary stage**	Localized	-
	Regional	1.71 (1.19-2.46; $p = 0.004$)
	Distant	4.57 (3.25-6.43; $p < 0.001$)

Concordance index (± standard error): 0.766 ± 0.009

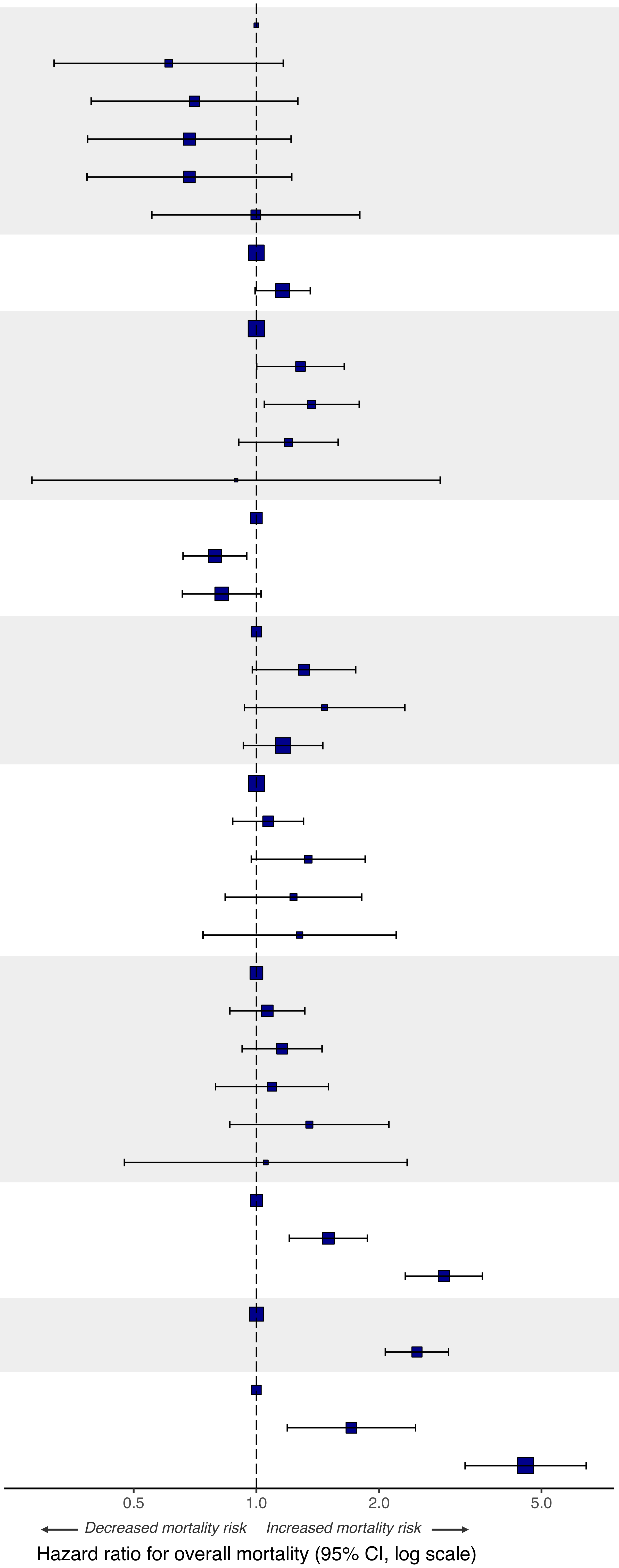

Supplementary Figure 2 – Multivariable Cox proportional hazards model for overall survival (OS), survival cohort
