# Supplementary material for: Predicting Survival in Mucinous Adenocarcinoma of the Appendix: Demographics, Disease Presentation, and Treatment Methodology
Source: Ann Surg Oncol. 2024 Jun 14;31(9):6237–51. doi: 10.1245/s10434-024-15526-z (PMC11300641; doi:10.1245/s10434-024-15526-z)
Supplement: Supplementary file 6 — Supplementary file6 Supplementary Fig. 3 Kaplan-Meier survival curves, cytoreductive surgery with hyperthermic intraperitoneal chemotherapy (CRS-HIPEC) rationale cohort: (a) disease-specific survival (DSS) of CRS-HIPEC vs. other surgery; (b) DSS of CRS-HIPEC vs. other surgery, by surgery/systemic therapy combination; (c) overall survival (OS) of CRS-HIPEC vs. other surgery; (d) OS of CRS-HIPEC vs. other surgery, by surgery/systemic therapy combination (925 KB) [file 10434_2024_15526_MOESM6_ESM.pdf]

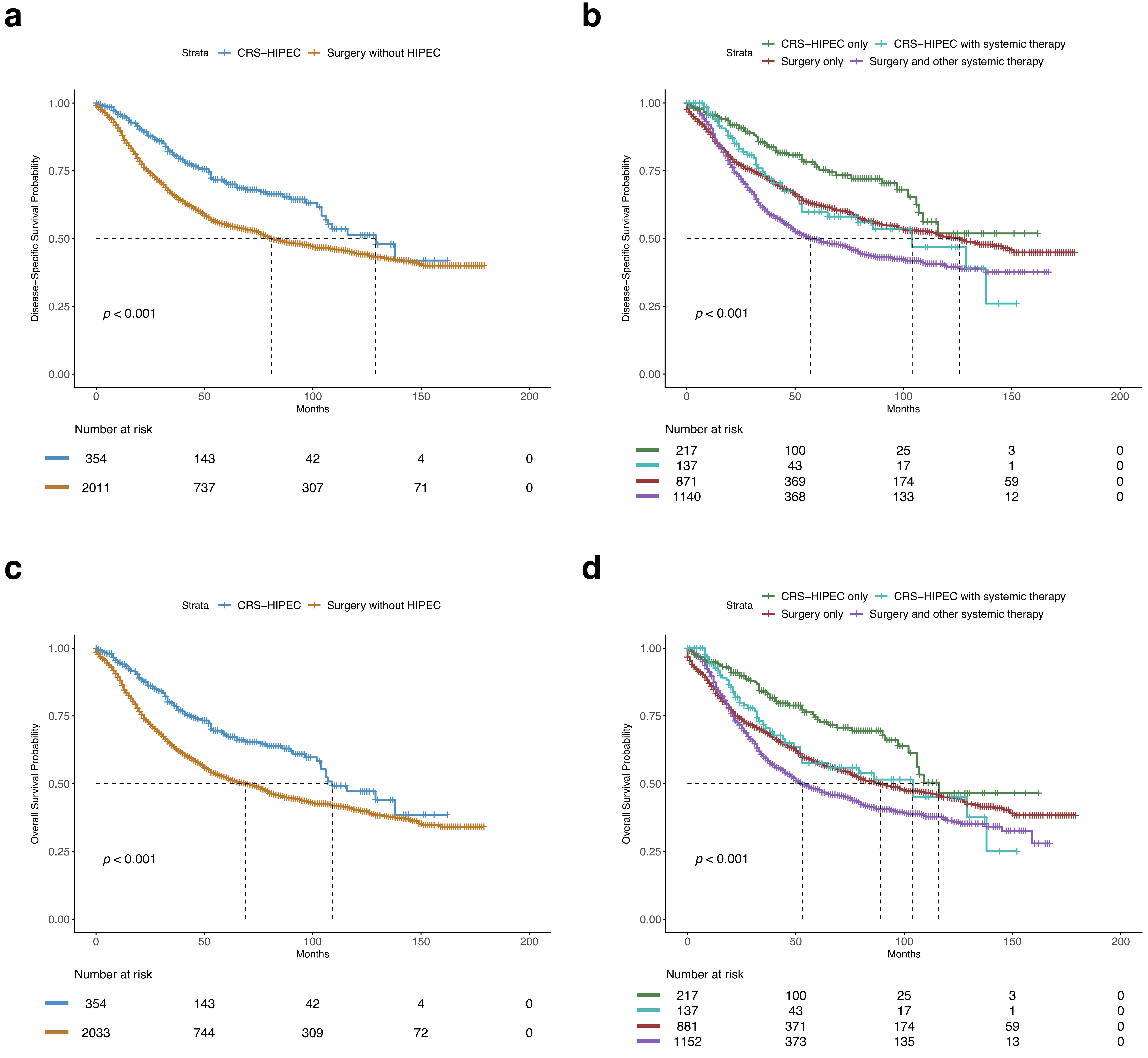

Supplementary Figure 3 – Kaplan-Meier survival curves, cytoreductive surgery with hyperthermic intraperitoneal chemotherapy (CRS-HIPEC) rationale cohort: **(a)** disease-specific survival (DSS) of CRS-HIPEC vs. other surgery; **(b)** DSS of CRS-HIPEC vs. other surgery, by surgery/systemic therapy combination; **(c)** overall survival (OS) of CRS-HIPEC vs. other surgery; **(d)** OS of CRS-HIPEC vs. other surgery, by surgery/systemic therapy combination
