# Supplementary material for: Predicting Survival in Mucinous Adenocarcinoma of the Appendix: Demographics, Disease Presentation, and Treatment Methodology
Source: Ann Surg Oncol. 2024 Jun 14;31(9):6237–51. doi: 10.1245/s10434-024-15526-z (PMC11300641; doi:10.1245/s10434-024-15526-z)
Supplement: Supplementary file 7 — Supplementary file7 Supplementary Fig. 4 Multivariable Cox proportional hazards model for overall survival (OS), cytoreductive surgery with hyperthermic intraperitoneal chemotherapy (CRS-HIPEC) rationale cohort (98 KB) [file 10434_2024_15526_MOESM7_ESM.pdf]

Variable	Cohort	HR (95% CI; *p*-value)
**Age, by decade**	20-29 years	-
	30-39 years	0.63 (0.32-1.23; $p = 0.176$)
	40-49 years	0.75 (0.41-1.37; $p = 0.347$)
	50-59 years	0.71 (0.39-1.30; $p = 0.264$)
	60-69 years	0.70 (0.38-1.28; $p = 0.250$)
	70-79 years	0.91 (0.49-1.69; $p = 0.771$)
**Sex**	Female	-
	Male	1.24 (1.05-1.46; $p = 0.010$)
**Race/ethnicity**	Non-Hispanic White	-
	Hispanic (All Races)	1.27 (0.98-1.65; $p = 0.070$)
	Non-Hispanic Black	1.33 (1.00-1.77; $p = 0.052$)
	Non-Hispanic Asian or Pacific Islander	1.20 (0.89-1.61; $p = 0.225$)
	Non-Hispanic Other Race	0.86 (0.27-2.72; $p = 0.796$)
**Year of diagnosis**	2005-2009	-
	2010-2014	0.91 (0.74-1.11; $p = 0.331$)
	2015-2019	0.95 (0.75-1.21; $p = 0.694$)
**U.S. Census region**	Northeast	-
	South	1.32 (0.97-1.80; $p = 0.075$)
	Midwest	1.49 (0.93-2.38; $p = 0.097$)
	West	1.14 (0.91-1.44; $p = 0.261$)
**Urban/rural classification**	Urban (pop. > 1,000,000)	-
	Urban (pop. 250,000-1,000,000)	0.98 (0.79-1.21; $p = 0.828$)
	Urban (pop. < 250,000)	1.33 (0.95-1.86; $p = 0.096$)
	Rural (urban-adjacent)	1.31 (0.89-1.94; $p = 0.176$)
	Rural	1.15 (0.65-2.02; $p = 0.633$)
**Median household income**	More than $75,000	-
	$65,000 - $74,999	1.09 (0.87-1.35; $p = 0.461$)
	$55,000 - $64,999	1.12 (0.88-1.42; $p = 0.361$)
	$45,000 - $54,999	1.06 (0.76-1.48; $p = 0.725$)
	$35,000 - $44,999	1.50 (0.94-2.38; $p = 0.090$)
	Less than $35,000	1.21 (0.54-2.72; $p = 0.647$)
**Histopathologic grade**	Well-differentiated - Grade 1	-
	Moderately differentiated - Grade 2	1.57 (1.24-1.98; $p < 0.001$)
	Poorly differentiated - Grade 3	3.19 (2.53-4.03; $p < 0.001$)
**Lymph node status**	Negative	-
	Positive	2.38 (1.98-2.86; $p < 0.001$)
**Combined summary stage**	Regional	-
	Distant	2.99 (2.46-3.64; $p < 0.001$)
**Time to treatment**	Less than 1 month	-
	1-2 months	0.98 (0.79-1.23; $p = 0.875$)
	2-3 months	0.94 (0.65-1.37; $p = 0.751$)
	More than 3 months	0.98 (0.61-1.59; $p = 0.946$)
**Treatment strategy**	Surgery only	-
	Surgery and other systemic therapy	0.74 (0.61-0.90; $p = 0.002$)
	CRS-HIPEC only	0.44 (0.30-0.64; $p < 0.001$)
	CRS-HIPEC with systemic therapy	0.60 (0.40-0.91; $p = 0.015$)

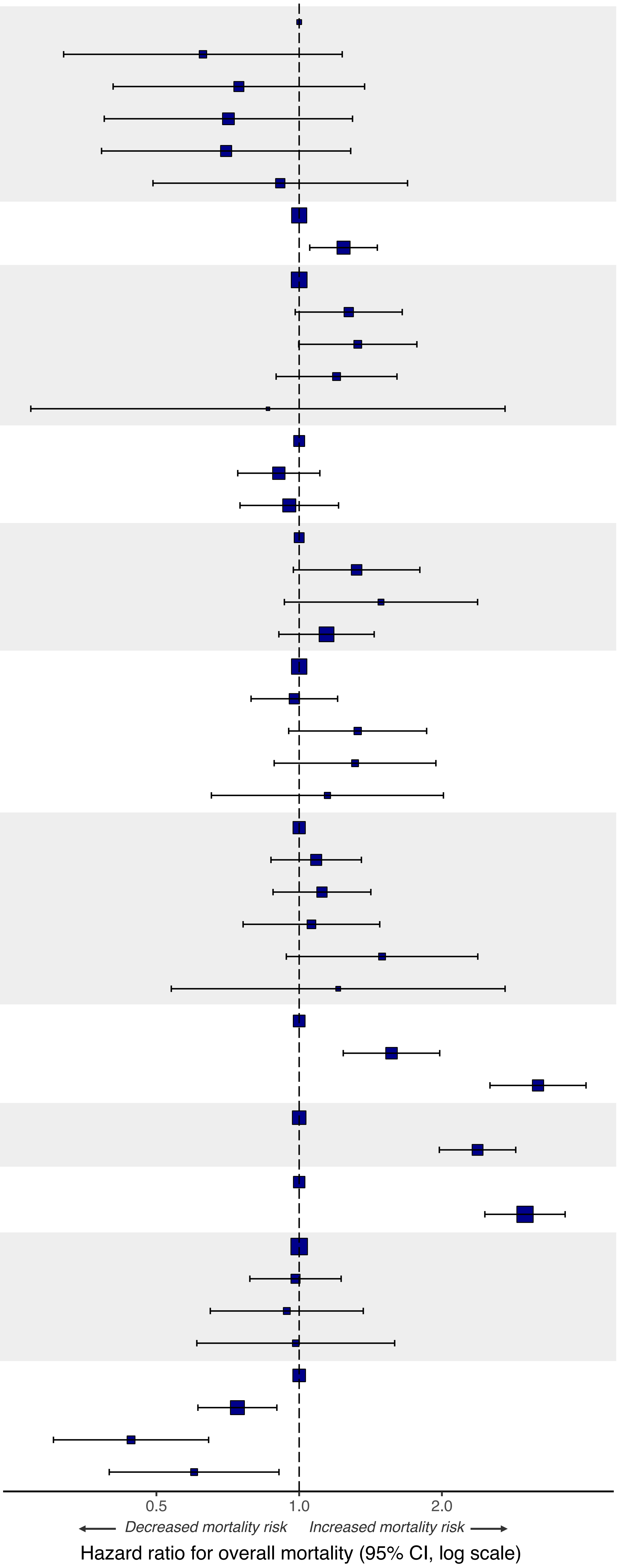

Concordance index (± standard error): 0.752 ± 0.010

Supplementary Figure 4 – Multivariable Cox proportional hazards model for overall survival (OS), cytoreductive surgery with hyperthermic intraperitoneal chemotherapy (CRS-HIPEC) rationale cohort
