# Supplementary material for: Predicting Survival in Mucinous Adenocarcinoma of the Appendix: Demographics, Disease Presentation, and Treatment Methodology
Source: Ann Surg Oncol. 2024 Jun 14;31(9):6237–51. doi: 10.1245/s10434-024-15526-z (PMC11300641; doi:10.1245/s10434-024-15526-z)
Supplement: Supplementary file 8 — Supplementary file8 Supplementary Fig. 5 Kaplan-Meier overall survival (OS) curves, cytoreductive surgery with hyperthermic intraperitoneal chemotherapy (CRS-HIPEC) rationale cohort, by grade and treatment strategy: (a) CRS-HIPEC vs. other surgery, grade 1 disease; (b) CRS-HIPEC vs. other surgery, grade 2 disease; (c) CRS-HIPEC vs. other surgery, grade 3 disease; (d) CRS-HIPEC vs. other surgery by treatment type, grade 1 disease; (e) CRS-HIPEC vs. other surgery by treatment type, grade 2 disease; (f) CRS-HIPEC vs. other surgery by treatment type, grade 3 disease (847 KB) [file 10434_2024_15526_MOESM8_ESM.pdf]

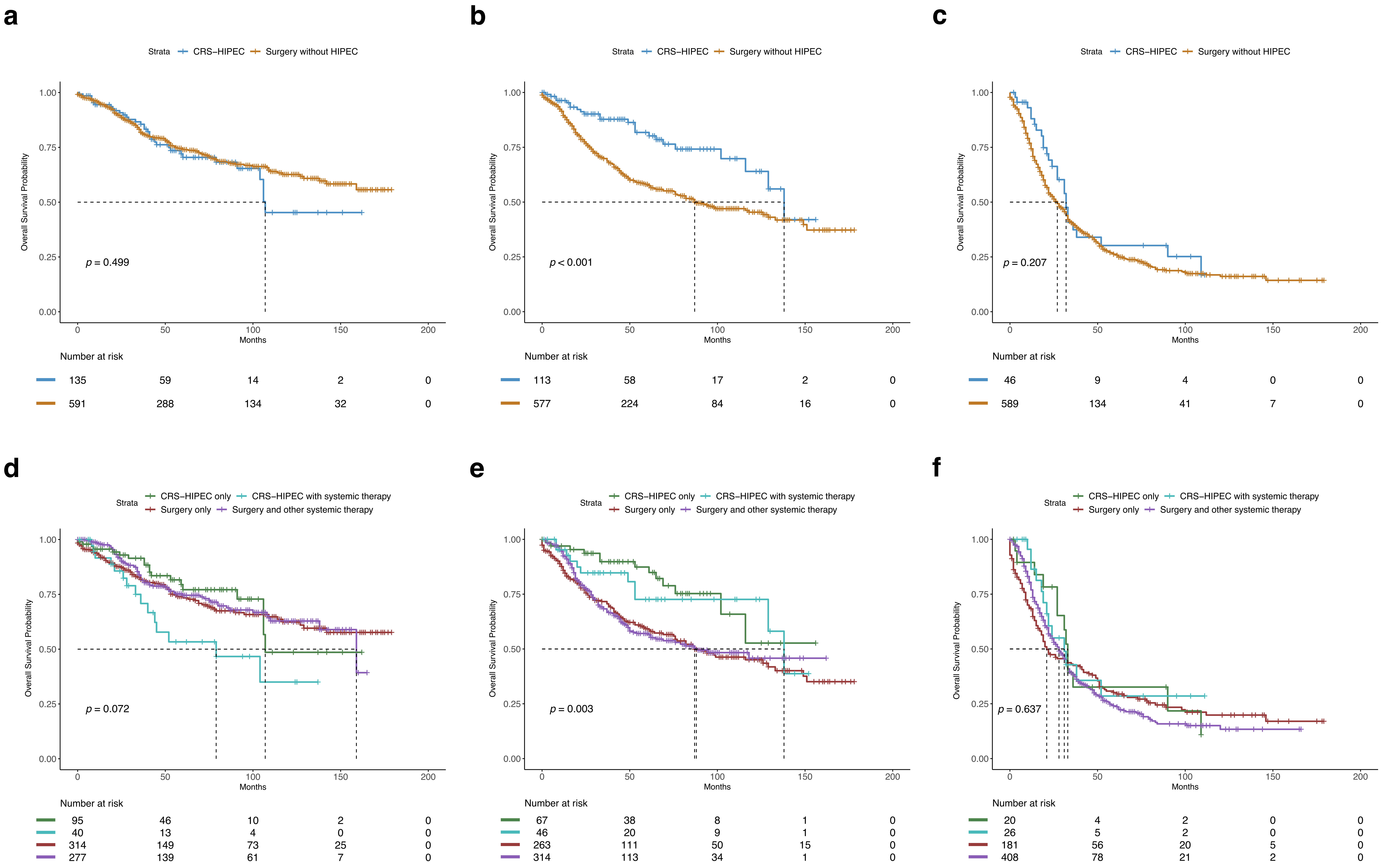

Supplementary Figure 5 – Kaplan-Meier overall survival (OS) curves, cytoreductive surgery with hyperthermic intraperitoneal chemotherapy (CRS-HIPEC) rationale cohort, by grade and treatment strategy: **(a)** CRS-HIPEC vs. other surgery, grade 1 disease; **(b)** CRS-HIPEC vs. other surgery, grade 2 disease; **(c)** CRS-HIPEC vs. other surgery, grade 3 disease; **(d)** CRS-HIPEC vs. other surgery by treatment type, grade 1 disease; **(e)** CRS-HIPEC vs. other surgery by treatment type, grade 2 disease; **(f)** CRS-HIPEC vs. other surgery by treatment type, grade 3 disease
